# Uncovering N4-Acetylcytidine-Related mRNA Modification Pattern and Landscape of Stemness and Immunity in Hepatocellular Carcinoma

**DOI:** 10.3389/fcell.2022.861000

**Published:** 2022-04-14

**Authors:** Sicheng Liu, Yaguang Zhang, Lei Qiu, Su Zhang, Yang Meng, Canhua Huang, Zhixin Chen, Bo Zhang, Junhong Han

**Affiliations:** ^1^ Research Laboratory of Cancer Epigenetics and Genomics, Department of General Surgery, Frontiers Science Center for Disease-Related Molecular Network, State Key Laboratory of Biotherapy and National Clinical Research Center for Geriatrics, West China Hospital, Sichuan University, Chengdu, China; ^2^ Department of Gastrointestinal Surgery, Frontiers Science Center for Disease-Related Molecular Network, West China Hospital, Sichuan University, Chengdu, China

**Keywords:** hepatocellular carcinoma, ac4C, mRNA modification, tumor microenvironment, tumor stemness, prognosis predictor

## Abstract

N4-acetylcytidine (ac4C) is an ancient and conserved RNA modification. Previously, ac4C mRNA modification has been reported promoting proliferation and metastasis of tumor cells. However, it remains unclear whether and how ac4C-related mRNA modification patterns influencing the prognosis of hepatocellular carcinoma (HCC) patients. Hereby, we constructed an ac4Cscore model and classified patients into two groups and investigated the potential intrinsic and extrinsic characteristics of tumor. The ac4Cscore model, including COL15A1, G6PD and TP53I3, represented ac4C-related mRNA modification patterns in HCC. According to ac4Cscore, patients were stratified to high and low groups with distinct prognosis. Patients subject to high group was related to advanced tumor stage, higher TP53 mutation rate, higher tumor stemness, more activated pathways in DNA-repair system, lower stromal score, higher immune score and higher infiltrating of T cells regulatory. While patients attributed to low group were correlated with abundance of T cells CD4 memory, less aggressive immune subtype and durable therapy benefit. We also found ac4Cscore as a novel marker to predict patients’ prognosis with anti-PD1 immunotherapy and/or mTOR inhibitor treatment. Our study for the first time showed the association between ac4C-related mRNA modification patterns and tumor intrinsic and extrinsic characteristics, thus influencing the prognosis of patients.

## Introduction

Worldwide, liver cancer has become a prominent public health problem with high morbidity and mortality. According to the latest statistics, liver cancer causes more than 9,00,000 cases, ranks sixth in terms of incidence and is the third leading cause of cancer-related death in 2020, leaving patients few treatment alternatives (Sung et al., 2021). Hepatocellular carcinoma (HCC) is a major histological subtype of liver cancer, accounting for 90% of primary liver cancer cases (Llovet et al., 2021). Notably, patients with HCC in China have a poor five-year survival rate of only 12% (Zheng et al., 2018). Various risk factors contribute to HCC tumorigenesis, including alcohol abuse, hepatitis B and C viral infection and metabolic disease (Yang J. D. et al., 2019). Traditional treatment options for HCC mainly rely on radiotherapy, chemotherapy, surgical resection and transplantation (Gao et al., 2021). Currently, targeted therapy, such as anti-mammalian target of rapamycin (mTOR) inhibitors, and immune checkpoint blockade therapy, such as anti-programmed cell death protein 1 (PD1) inhibitors, have great potential effects against HCC (Ruiz de Galarreta et al., 2019; Wang et al., 2019). However, due to the complicated pathogenesis, rapidly proliferating activities, unrevealed mechanism of invasion, migration and insensitive response, tumor relapse and metastasis subsequently occur after treatment, resulting in limited clinical benefit and poor prognosis of patients. Therefore, it is particularly urgent to discover and identify novel HCC markers for early diagnosis, prediction of prognosis, and to explore the molecular mechanisms behind the process of tumor initiation and progression. Thus, healthcare burden should be relieved, and the life quality of patients should be improved.

Cancer stem cells (CSCs) represent a rare subpopulation of cancer cells that exhibit extensive features of normal stem cells, such as limitless division, self-renewal and differentiation. Growing evidence has demonstrated that tumor recurrence, metastasis and the development of drug resistance can be explained by the presence of CSCs, leading to poor prognosis of patients (Visvader and Lindeman, 2008; Schulenburg et al., 2015). Recent studies have identified liver CSCs with several marker genes, including prominin 1 (PROM1), CD44, epithelial cell adhesion molecule (EPCAM) and others (Sun et al., 2016). Moreover, researchers have managed to identify tumor stemness characteristics using machine learning algorithm through transcriptome sequencing, promoting our understanding of CSCs and their roles and connections to specific oncogenic signaling pathways that sustain tumor growth and proliferation (Malta et al., 2018). In addition to CSCs, the tumor microenvironment (TME) consists of various stromal and immune cells, which together coregulate the progression of tumors and therefore influence the therapeutic response and prognosis of patients (Binnewies et al., 2018). For instance, differences in the composition of macrophages, neutrophils and T cells are correlated with clinical outcomes (Gonzalez et al., 2018). Moreover, the abundance of infiltrating T regulatory cells suppresses anticancer immunity, resulting in drug resistance (Togashi et al., 2019). With computational tools such as ESTIMATE and CIBERSORT, the TME of tumor samples can be reasonably measured at low cost, providing new insight for clinical research (Yoshihara et al., 2013; Newman et al., 2015).

Cancer is well accepted as a heterogeneous disease caused by the accumulation of genetic and epigenetic variations. For example, BAZ2A directly interacts with EZH2 to maintain epigenetic silencing at genes repressed in prostate cancer metastasis (Gu et al., 2015). Additionally, down-regulated expression level of IRS-1 mediated by miRNA-203 leads to significant decrease of metastasis and proliferation capacities of prostate cancer cells (Meng et al., 2020). And new technologies had advanced the study of associations between diseases and epigenetics (Wang et al., 2013). Nevertheless, emerging evidence has revealed that posttranscriptional RNA modification, also termed the epitranscriptome, participates in the complex biological process of tumorigenesis, invasion, metastasis and even drug resistance (Delaunay and Frye, 2019). N6-methyladenosine (m6A), accounting for the most abundant mRNA modification, has been widely studied since debut. Based on the large number of m6A regulators being determined thus far, comprehensive bioinformatic analyses revealed that m6A modification patterns were involved in TME infiltration of gastric cancer (Zhang et al., 2020), clear cell renal cell carcinoma (Zhong et al., 2021) and pancreatic cancer (Zhou et al., 2021) and therefore are associated with the response and prognosis of patients treated with immunotherapy. In contrast, there are more than 170 RNA modifications whose functions in cancer are barely known yet (Esteve-Puig et al., 2020). N4-acetylcytidine (ac4C) modification is an ancient and highly conserved RNA modification previously reported in rRNAs and tRNAs (Jin et al., 2020), whereas the latest research found that ac4C modifications catalyzed by N-acetyltransferase 10 (NAT10) in human could also occur on a broad range of mRNAs in a dynamic fashion, promoting the translation process and enhancing mRNA stability (Arango et al., 2018; Sas-Chen et al., 2020). In Arango’s study, NAT10-ablated HeLa cells showed reduced proliferative ability in an ac4C-dependent manner. Zhang et al. found that ac4C of COL5A1 mediated by NAT10 promoted metastasis of gastric cancer (Zhang et al., 2021). These results again confirmed the pivotal roles of ac4C mRNA modification in cancer progression. Therefore, comprehensive recognition of the association between intrinsic and extrinsic characteristics of tumor and ac4C modification patterns will contribute to our understanding of tumor management and treatment.

In this study, we identified differentially expressed genes between tumor and normal liver samples that are modified by NAT10-mediated ac4C and determined 21 genes. Combined with multivariate Cox regression and least absolute shrinkage and selection operator (LASSO) algorithms, COL15A1, G6PD and TP53I3 were selected to construct the risk model, termed ac4Cscore. We also demonstrated that ac4Cscore could classify patients into two groups with distinct prognoses, pathologies and somatic mutation profiles. The association between ac4Cscore and tumor stemness as well as TME infiltration was further confirmed using bioinformatic tools. Since ac4C is a conserved modification, we proved that ac4Cscore was applicable not only in the prediction of stemness, TME distribution and survival outcome of patients with HCC, but also in those with lung and pancreatic cancers. Finally, we validated the practical value of ac4Cscore using clear cell renal cell carcinoma cohorts treated with anti-PD1 and anti-mTOR inhibitors. These results illustrated that ac4Cscore was a promising biomarker to predict the prognosis of patients.

## Materials and Methods

### Dataset and Preprocess

Three publicly available databases were collected in this study, including The Cancer Genome Atlas (TCGA, https://portal.gdc.cancer.gov/), International Cancer Genome Consortium (ICGC, https://dcc.icgc.org/) and Gene Expression Omnibus (GEO, https://www.ncbi.nlm.nih.gov/geo/). For TCGA database, normalized gene expression data of liver hepatocellular carcinoma (LIHC), pancreatic adenocarcinoma (PAAD) and lung adenocarcinoma (LUAD) by log_2_(FPKM+1) were downloaded from UCSC Xena (https://xenabrowser.net/datapages/) along with clinical information (Goldman et al., 2020). The “ComBat” function from the R package sva was used to alleviate batch effects and to keep biological differences (Leek et al., 2012). Matched single nucleotide variants (SNVs), insertion-deletion variants (INDELs) and copy number variants (CNVs) of patients were extracted from the Genomic Data Commons (GDC). For the ICGC database, normalized expression data in FKPM format along with survival information from liver cancer patients (LIRI) were extracted (ICGC/TCGA Pan-Cancer Analysis of Whole Genomes Consortium, 2020). For the GEO database, the R package GEOqurey was used to acquire Affymetrix microarray gene expression data normalized by the RMA method and matched phenoData of human hepatocellular carcinoma (GSE14520) (Davis and Meltzer, 2007; Roessler et al., 2010). Immune blockade therapy (anti-PD1) and mTOR inhibitor treatment (anti-mTOR) in a clear cell renal cell carcinoma cohort were also enrolled in this study. Whole-exome and transcriptome data were obtained from supplementary tables of the published article (Braun et al., 2020). To make it more comparable among multiple cohorts and similar to microarray data, RNA-sequencing data were transformed into transcripts per kilobase million (TPM) values based on the protein-coding gene annotations in GENCODE v22 and then logarithmically scaled. Dataset summaries are listed in Supplementary Table S1. Immunohistochemistry was obtained from The Human Protein Atlas (https://www.proteinatlas.org/). The tumor immune dysfunction and exclusion (TIDE) method, a well-known biomarker for modeling two primary mechanisms of tumor immune evasion and predicting the response of patients, was used in the anti-PD1 cohort (Jiang et al., 2018). TIDE scores were calculated with an online website (http://tide.dfci.harvard.edu).

### Identification of Differentially Expressed ac4C-Modified Genes

The empirical Bayesian method from the R package limma (v3.42.2) was performed to identify differentially expressed genes of tumor and normal livers in LIHC with the threshold |log_2_FC |> 2 and *p*-value < 0.05 (Ritchie et al., 2015). Considering that ac4C is an ancient and highly conserved modification in eukaryotic mRNA, and NAT10 is the only known acetyltransferase at present, a list of ac4C-modified genes was selected manually from supplementary tables of the published article, which conducted ac4C-RIP-seq to screen modified peaks using wild-type and NAT10-ablated HeLa cells (Arango et al., 2018). Raw sequencing data were downloaded from the Gene Expression Omnibus dataset (GSE102113). Reads were first trimmed to remove low-quality bases and adaptor sequences using Trimmomatic (version 0.39) (Bolger et al., 2014). Hisat2 (version 2.2.1) was then used to map the reads to the human genome (hg38) (Kim et al., 2019). Samtools (version 1.9) was used to sort the coordinates of reads (Li et al., 2009). The BamCoverage function from deepTools (version 3.5.0) was applied to normalize aligned data (Ramirez et al., 2016). Finally, sample files were exported and visualized in IGV (version 2.8.10) (Thorvaldsdottir et al., 2013). Gene lists were intersected and defined as ac4C-DEGs for further analysis.

### Construction and Validation of ac4C Gene Signature

Both multivariate Cox regression model and LASSO with 10-fold cross-validation algorithm were used to select the ac4C-DEGs that were significantly associated with prognosis in LIHC (Simon et al., 2011). The results were determined and visualized by the R package glmnet (v 4.1–1) and forestplot (1.10.1). Ac4C-DEGs with a *p*-value < 0.05 in the multi-Cox model and included in LASSO were considered key genes and validated using univariate Cox regression model based on the R packages survival (v3.2–7) and survminer (v0.4.8). Key genes were first scaled and standardized by Z-score. Then, ac4Cscore was calculated by the following formula: $ac4Cscore=\overset{n}{\underset{i}{\sum }}Exp_{i\;}\text{* }Coef_{i}$ . where $Exp_{i\;}$ is the scaled expression and $Coef_{i}$ is the coefficient of each key gene. This method ensures the comparability of ac4Cscore in different cohorts measured by various platforms. In addition, the maximum selected rank statistics method using the surv_cutpoint function from the R package survminer was applied to stratify patients into high and low ac4Cscore groups, thus reducing the batch effect of different cohorts. The prognostic value of ac4Cscore was further examined in other datasets using the Kaplan-Meier method with the log-rank test.

### Somatic Mutation Analysis

SNVs and INDELs of ac4C-DEGs in LIHC and high-ac4Cscore and low-ac4Cscore groups in the mentioned datasets were analyzed to profile the variant characteristics. The capture size of the whole exon was deliberately set as 40 for calculation of tumor mutation burden (TMB) because it would not affect the result. Mutant-allele tumor heterogeneity (MATH) was calculated by univariate density and cluster classification based on the variant allele frequency of individuals. All approaches above were performed by R package maftools (v2.6.05) (Mayakonda et al., 2018). Genomic Identification of Significant Targets in Cancer 2 (GISTIC2) software was used to detect CNV changes in the two ac4Cscore groups of LIHC (Mermel et al., 2011). The parameters were set as -brlen 0.5 -conf 0.90. UCSC hg38 was used as reference genome. Significantly aberrant regions were determined by *q*-value < 0.25.

### Functional Enrichment Analysis

Gene Ontology (GO) and Kyoto Encyclopedia of Genes and Genomes (KEGG) analyses of integrated ac4C-DEGs were performed using the R package clusterProfiler (v3.14.3) (Yu et al., 2012). Cutoffs of *p*-value < 0.05 and *q*-value < 0.2 indicated statistical significance.

Gene set enrichment analysis (GSEA) was performed to determine the concordant biological differences between the ac4Cscore-high and ac4Cscore-low groups in three HCC datasets (Subramanian et al., 2005). The R package limma was used to calculate the fold changes. Gene list was sorted by decreasing value. Gene sets c2.all.v7.4.symbols.gmt downloaded from the Molecular Signatures Database (MSigDB) and six categories of pathway information from KEGG (https://www.genome.jp/kegg/) were utilized as references in the R package clusterProfiler. A strict criterion with *p*-value < 0.01 and adjusted *p*-value < 0.05 represented statistical significance.

### Estimation of Tumor Stemness

Cancer stem cells possess the abilities of self-renewal and differentiation, thus potentially giving rise to tumor progression and metastasis. A list of embryonic and liver cancer stem cell markers was obtained by comprehensive literature retrieval. Tathiane M. Malta et al developed an innovative one-class logistic regression (OCLR) machine learning algorithm to reveal characteristics of tumor stemness (Malta et al., 2018). The estimated cancer mRNA expression-based stemness index (mRNAsi) for TCGA datasets were extracted from the supplementary table of the paper. For other datasets, mRNAsi for each patient was predicted by the workflow described in this paper.

### Estimation of Tumor Microenvironment

A variety of tumor and nontumor cells were mixed within the tumor microenvironment, which influenced tumor progression. In this context, the ESTIMATE algorithm was used to predict tumor purity and the presence of stromal and immune cell infiltration (Yoshihara et al., 2013). In addition, the CIBERSORT algorithm, which is based on a novel application of nu-support vector regression, was performed to estimate the relative fraction of 22 immune cell types with bulk transcriptome data from tumor tissues with parameter permutation test set as 1,000 times (Newman et al., 2015). The immune subtypes of individual LIHC patients were obtained from a previously published paper (Thorsson et al., 2018). To further examine the correlation of ac4Cscore and immune-related pathways, gene set variant analysis from the R package GSVA was applied to evaluate the variation in pathway activity over the ac4C-high and ac4C-low groups. A list of immune signatures was downloaded from the Immunology Database and Analysis Portal (ImmPort, https://www.immport.org/shared/genelists).

### Cell Culture and Gene Knockdown

HepG2, Huh7 and 293T cell lines verified by STR profiling were purchased from National Collection of Authenticated Cell Cultures (Shanghai, China) and maintained in DMEM (HyClone, AG29629575) supplemented with 10% FBS (ExCell Bio, FSP500) and 100 U/ml penicillin/streptomycin (Beyotime, C0222) at 37°C in a humidified incubator (Thermo) with 5% CO2.

Gene knockdown was achieved using the short hairpin RNA system. The pLKO.1 vector was digested with AgeI (NEB, R3552) and EcoRI (NEB, R3101). Oligos were synthesized as follow: shNAT10#1, forward primer: CCG​GTT​GCT​GTT​CAC​CCA​GA-TTA​TCC​TCG​AGG​ATA​ATC​TGG​GTG​AAC​AGC​AAT​TTT​TG, reverse primer: AAT​T-CAA​AAA​TTG​CTG​TTC​ACC​CAG​ATT​ATC​CTC​GAG​GAT​AAT​CTG​GGT​GAA​CAG-CAA; shNAT10#2, forward primer: CCG​GGA​GAT​GTA​TTC​ACG​GAA​TAT​GCT​CG-AGC​ATA​TTC​CGT​GAA​TAC​ATC​TCT​TTT​TG, reverse primer: AAT​TCA​AAA​AGA​G-ATG​TAT​TCA​CGG​AAT​ATG​CTC​GAG​CAT​ATT​CCG​TGA​ATA​CAT​CTC. The forward oligo and reverse oligo were phosphorylated, annealed and further ligated to the linearized pLKO.1 vector by T4 DNA ligase (NEB, M0202). 293T cells were transfected with pLKO.1-shNAT10 with packing plasmids psPAX2 and PMD2.G. The resulting viral supernatant was used to infect target cells. Infected cells were selected with 2 μg/ml puromycin (InvivoGen).

### Cell Proliferation Assay

Cell proliferation assays were performed as previously described (Liu et al., 2021). For CCK-8, a total of 2,000 cells were seeded in 96-well plates, and Cell Counting Kit-8 (CCK-8, TargetMol, C0005) was added and incubated for 3 h at days 0, 1, 2, 3, and 4 after seeding. The absorbance was measured with a microplate reader (BioTek) at 450 nm.

For colony formation, a total of 2,000 cells were seeded in 24-well plates and cultured for 10–14 days to form macroscopic clones. The colony was fixed with 4% paraformaldehyde for 20 min, washed twice with PBS buffer, and stained with crystal violet solution at room temperature for 30 min. Colony number was counted after washing with ddH_2_O.

### EdU Staining Assay

EdU was detected using a Cell-Light Edu Apollo488 *in Vitro* Kit (RiboBio) according to manufacturers’ protocol. Briefly, control (shNT) and NAT10 knockdown (shNAT10_1# and shNAT10_2#) cells were seeded onto coverslips in 24-well. Cells were incubated with EdU solution (50 μM) for 2 h. 4% paraformaldehyde was fixed for 30 min, neutralized with 2 mg/ml glycine solution, incubated with penetrant (0.5% Triton X-100 in PBS). Apollo solution stained for 30 min in dark, washed twice with penetrant and methanol, counterstained the cell with Hoechst33342. Images were captured using fluorescence microscope (Nikon).

### Apoptosis Assay

Apoptosis assay was detected using an Annexin V-FITC/PI Kit (KeyGen BioTECH) according to manufacturers’ protocol. Briefly, cells (shNT, shNAT10_1# and shNAT10_2#) were collected after trypsinization, washed twice with pre-cooled PBS, and resuspended in binding buffer to adjust the cell concentration to 5 × 10^6/ml. Take 100 μl of cell suspension and add 5 μl of Annexin V/FITC to incubate for 5 min, add 10 μl of PI and 400 μl of PBS, and then use flow cytometer (Beckman) for detection.

### Statistical Analysis

R software 3.6.3 (https://www.R-project.org/) and GraphPad Prism five were used for statistical analysis. Student’s t test was applied to calculate the *p*-value of continuous variables. When data were not normally distributed, the Wilcoxon test was used. Fisher’s exact test was performed to compare categorical variables. Log-rank test was used for survival analysis. Pearson correlation coefficients were used to compare the relationship of two variables. A two-sided *p*-value < 0.05 was considered statistically significant unless otherwise specified. The R package pheatmap (v1.0.12) was used to scale and visualize the expression of ac4C-DEGs between tumor and normal tissues, and stemness-related genes and score of immune-related pathways between the ac4Cscore-high and ac4Cscore-low groups. The R package ggalluvial (v0.12.3) was used to visualize the attribution of immune subtype to individual patients in different ac4Cscore groups. The R package corrplot (v0.84) was applied to draw the correlation of ac4Cscore and immune signatures. The R package circlize (v0.4.14) was utilized for ring heatmap (Gu et al., 2014). The R package ggradar (v0.2) was used to display the distribution of immune cells. The R package timeROC (v0.4) was used for receiver operating characteristic analysis and to quantify the area under the curve.

## Results

### N-Acetyltransferase 10 Is Highly Expressed in Hepatocellular Carcinoma and Functions as a Potential Oncogene

**FIGURE 1:**
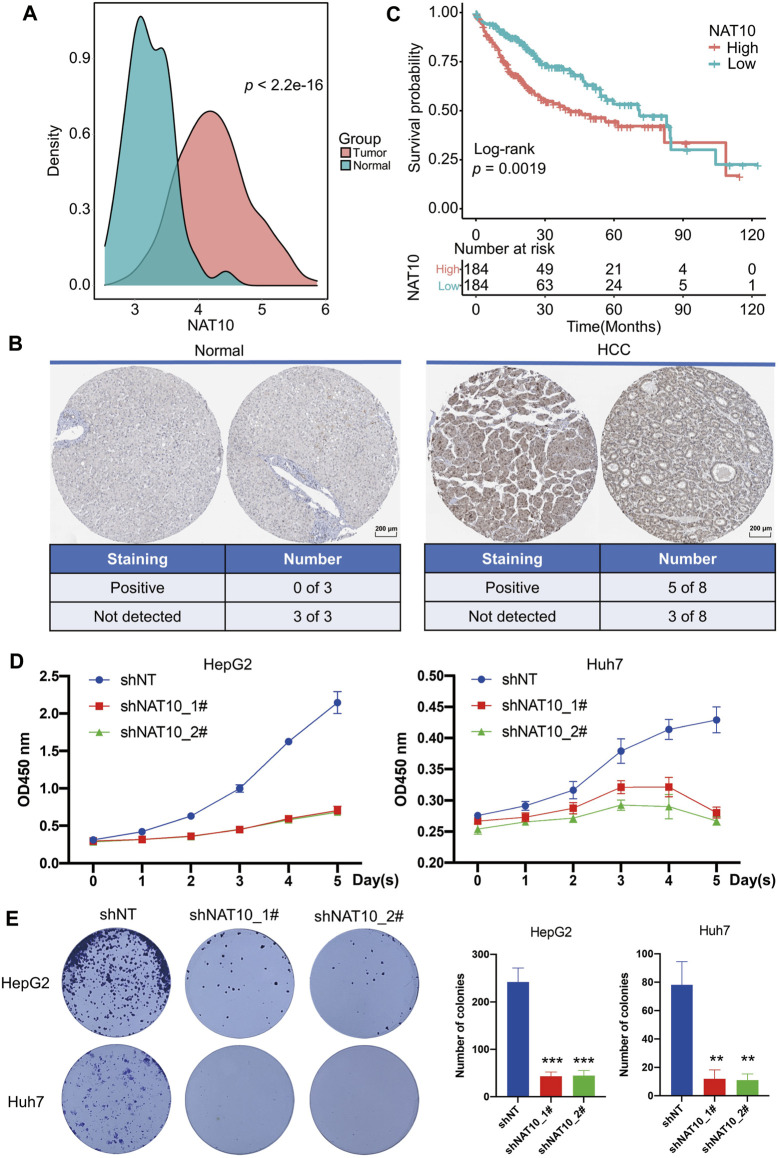
Potential oncogenic role of NAT10 in HCC. **(A)** Density plots show the expression of NAT10 in normal and HCC samples. Wilcox test with *p* value < 0.05 is considered as significant. **(B)** Representative images and statistics of IHC staining for NAT10 in liver tissues and HCC from the Human Protein Atlas dataset. Scale bar, 200 μM. **(C)** Survival analysis of NAT10 in HCC patients. Kaplan-Meier curves with log-rank *p* values < 0.05 are considered significant. **(D)** Cell proliferation of HepG2 and Huh7 cells after NAT10 knockdown measured with CCK-8, *n* = 3, data were expressed as mean ± SEM. **(E)** Cell proliferation of HepG2 and Huh7 cells after NAT10 knockdown measured with colony formation, *n* = 3. Left, representative image. Right, quantitative analysis, **, *p* < 0.01, ***, *p* < 0.001.

The expression of NAT10 in HCC and normal liver samples were downloaded from TCGA. NAT10 was aberrantly higher expressed in HCC compared with normal samples (Figure 1A). The protein expression of NAT10 was also examined using the Human Protein Atlas database, and the same trend was observed. Among eight HCC samples, five were staining-positive, while all three out of three normal liver samples were negative (Figure 1B). For those who diagnosed with HCC, higher expression of NAT10 indicated a shorter survival outcome, especially within the first 5 years (Figure 1C). To investigate cell proliferation in HCC cells lacking NAT10, CCK-8, colony formation and EdU staining assay were performed. Results showed that cell growth was significantly inhibited in HepG2 and Huh7 with NAT10 knocked down by shRNAs (Figures 1D,E and Supplementary Figure S1A–C). Interestingly, we also found that NAT10 might play a role in regulating cell apoptosis (Supplementary Figure S1 D,E). These results suggest NAT10 exerts a potential oncogenic role in HCC.

### Identification of N-Acetyltransferase 10-Related ac4C-DEGs in Liver Hepatocellular Carcinoma

**FIGURE 2:**
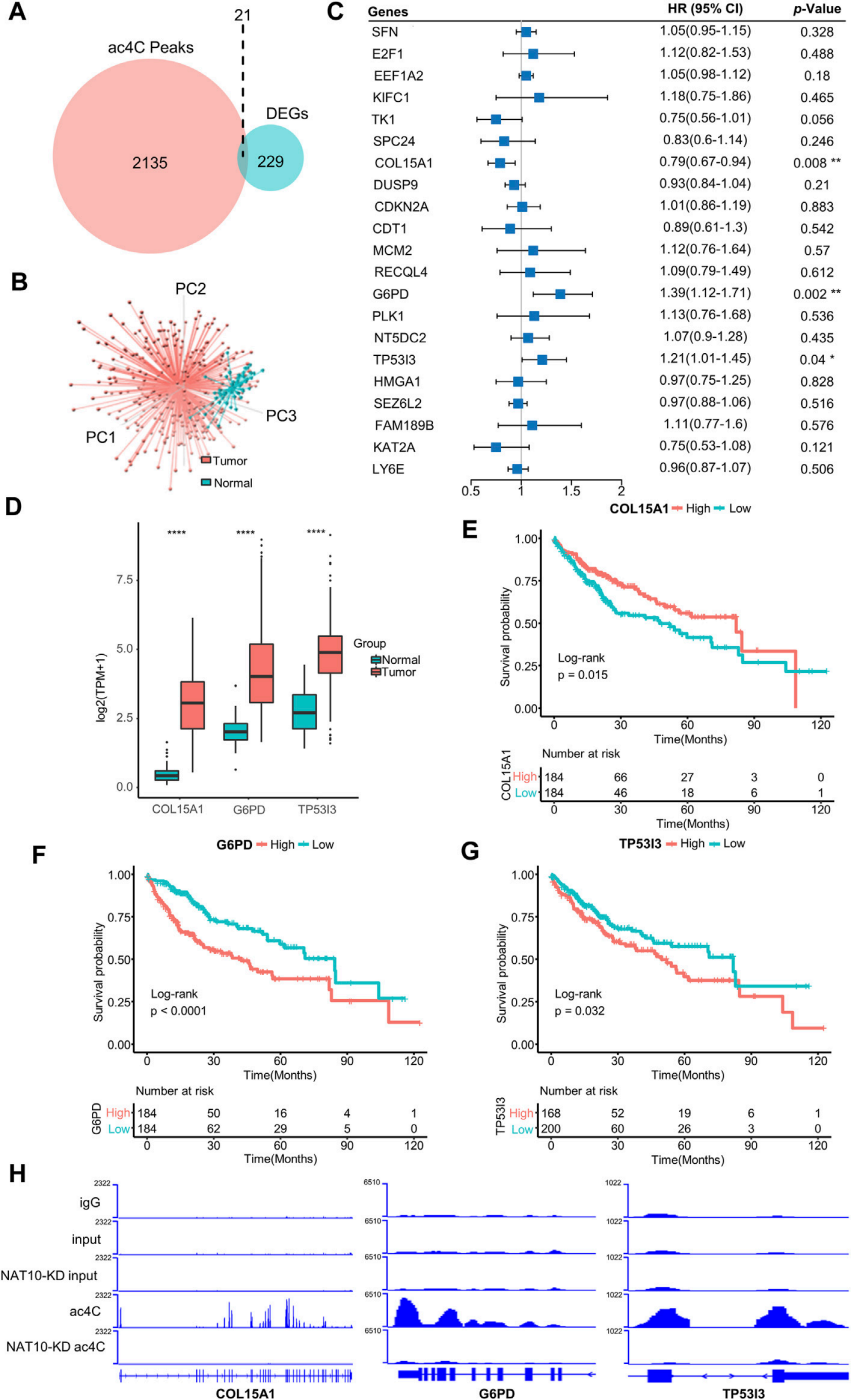
Identification of ac4C-DEGs associated with prognosis in HCC. **(A)** Venn diagram shows the number of intersected genes between ac4C peaks and DEGs. **(B)** Principal component analysis of the expression patterns of 21 ac4C-DEGs distinguishes tumor and normal samples. **(C)** Forest plot of multivariate Cox analysis determines three key genes significantly associated with overall survival. HRs are shown with 95% confidence intervals. * indicates *p* < 0.05 and ** *p* < 0.01. **(D)** The expression of three key genes between normal and tumor samples. The top and bottom of the boxes represent the 75th and 25th percentiles, respectively. The middle lines in the boxes represent median values. The black dots indicate outliers. **** indicates *p* < 0.0001. **(E–G)** Survival analysis for the three key genes. Kaplan-Meier curves with log-rank *p* values < 0.05 are considered significant. **(H)** Bar graphs show differential ac4C modification of COL15A1, G6PD, and TP53I3 between wild-type and NAT10-ablated cells.

NAT10 was known as an acetyltransferase to many molecular components. Recent study revealed the ability of NAT10 for mRNA acetylcytidine, bringing us new insight into epitranscriptome. A list of 2,156 genes that are modified by NAT10-mediated ac4C was obtained (Supplementary Table S2). A total of 250 genes showing significantly different expression patterns between tumor and normal liver tissues were identified (Supplementary Table S3). In these two gene sets, 21 genes intersected and were defined as ac4C-DEGs (Figure 2A). Most of these genes were upregulated in tumors compared with normal samples, while only *LY6E* was downregulated (Supplementary Figure S2A). Therefore, distinct expression patterns of these two groups could be observed with principal component analysis (Figure 2B). GO analysis demonstrated that ac4C-DEGs were associated with DNA replication and the cell cycle, while KEGG annotated that these genes were involved in the p53 signaling pathway and multiple tumor types (Supplementary Figure S2B). Somatic mutation analysis revealed that ac4C-DEGs had little SNV or INDEL (Fig S3A), but CNV results showed that the amplification frequencies of *FAM189B*, *LY6E*, *RECQL4* and *TK1*, and the deletion frequencies of *CDKN2A* and *SFN* were greater than 10% (Supplementary Figure S3B). Then, a multivariate Cox regression model and LASSO algorithm were constructed to determine the key genes that affected the prognosis of patients, and *COL15A1*, *G6PD* and *TP53I3* were selected (Figure 2C and Supplementary Figure S4A–C). Interestingly, while three key genes were significantly upregulated in tumor samples (Figure 2D), *COL15A1* played a role as a favorable factor, while the other two served as risk factors (Figure 2C). The independent prognostic value of the three key genes was further confirmed by K-M curve analysis (Figures 2E–G). Finally, the existence of ac4C modification mediated by NAT10 within *COL15A1*, *G6PD* and *TP53I3* was confirmed (Figure 2H). These results imply that ac4C-related modification genes are potentially involved in tumor initiation and are related to different prognoses of patients.

### Construction and Clinical Relevance of ac4Cscore

**FIGURE 3:**
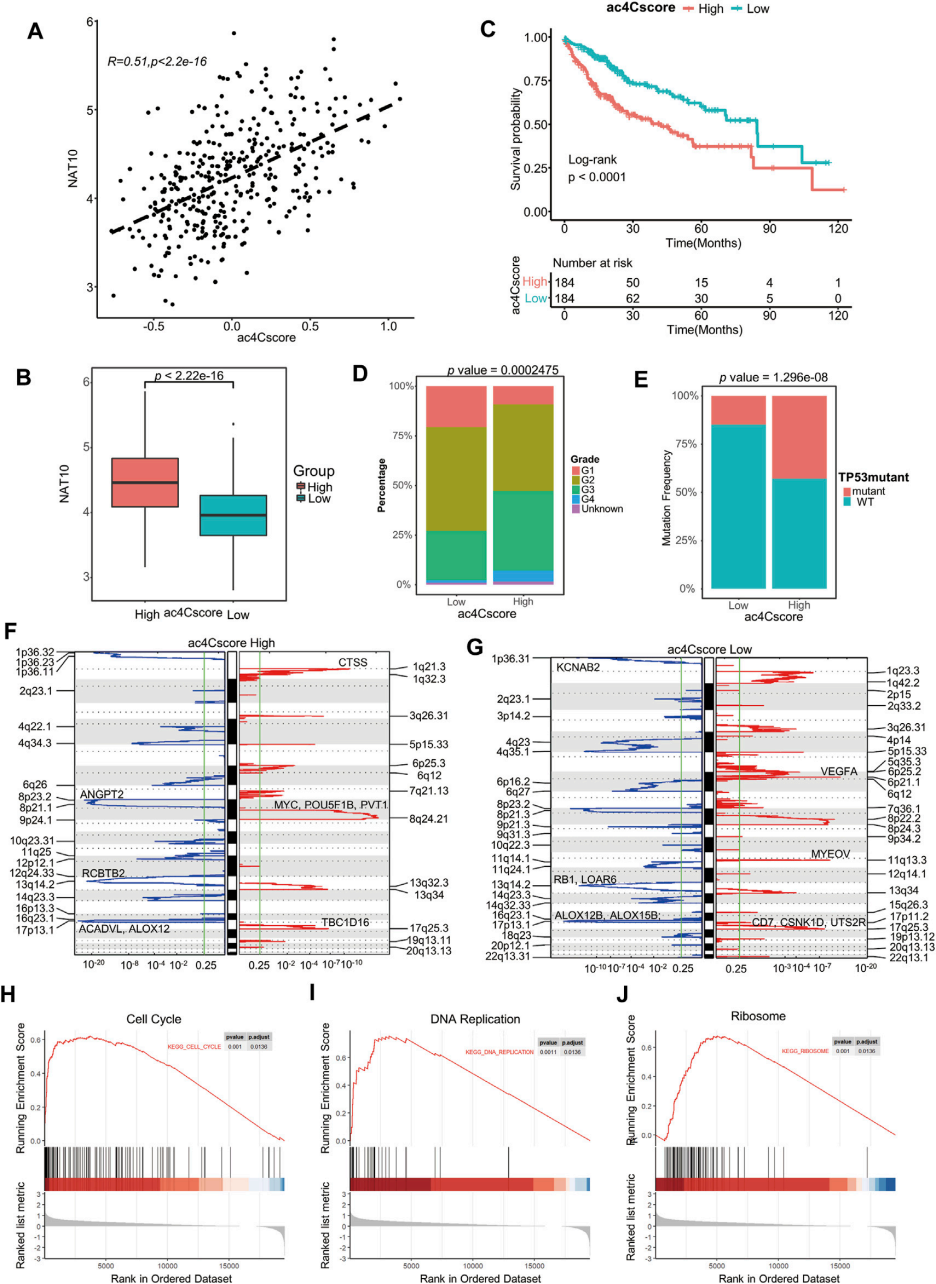
Construction of the ac4Cscore model and different biological characteristics of groups. **(A)** Correlation of ac4Cscore and expression of NAT10. Pearson correlation coefficient with *p* value < 0.05 is considered significant. **(B)** The expression of NAT10 between the ac4Cscore high and low groups. T test with *p* value < 0.05 is considered significant. **(C)** Survival analysis for ac4Cscore groups. Kaplan-Meier curves with log-rank *p* values < 0.05 are considered significant. **(D)** Proportion of tumor grade in the ac4Cscore groups. **(E)** Proportion of patients with or without *TP53* mutations in the ac4Cscore groups. Fisher’s exact test with *p* value < 0.05 is considered significant. **(F, G)** Significantly altered CNV regions of ac4Cscore high and low groups determined by GISTIC2. The left two panels show the profiles of the ac4Cscore high group, and the right two panels show the profiles of the low group. The panel with the red line indicates amplification regions, and the blue line indicates deletion regions. **(H–J)** GSEA plots show the upregulated genes in the ac4Cscore high group enriched in the cell cycle, DNA replication and ribosome pathways. A strict criterion with *p* value < 0.01 and adjusted *p* value < 0.05 is considered significant.

To profile the dynamic mRNA ac4C modification is costly, however, based on the knowledge that NAT10 is the only identified mRNA ac4C writer, the modification level should be positive correlated with the expression of NAT10, and the coefficient should not perfectly near to one because there might be other ac4C regulators not determined yet. Therefore, we constructed a model, termed ac4Cscore, using the expression and coefficient of key genes. Patients were divided into high and low groups according to their ac4Cscore (Supplementary Table S4). As expected, ac4Cscore was moderately positive correlated with the expression of NAT10 (Figure 3A), and high ac4Cscore group had significantly higher expression of NAT10 than that of low ac4Cscore group (Figure 3B). Unsurprisingly, those who had higher ac4Cscore suffered from significantly worse prognosis (Figure 3C). No age or gender differences were observed in these two groups since this was an unbiased model based only on transcriptome expression data (Supplementary Figures S5A,B). However, compared with the ac4C-low group, the ac4C-high group had a larger proportion of patients with advanced tumor grade (G3-4), suggesting the predictive power of ac4Cscore in clinical and pathological diagnosis (Figure 3D). There was no significant difference between the two groups of T, N and M stages (Supplementary Figures S5C–E).

The profile of somatic mutations between the low and high ac4Cscore groups was also analyzed. The high ac4Cscore group was notably associated with a higher prevalence of *TP53* mutations (Figure 3E). However, the TMB and MATH scores of these two groups were not significantly different (Supplementary Figures S5F,G). Therefore, GISTIC2 was used to detect most different aberrant CNV regions. The high ac4Cscore group contained fewer regions of both amplification and deletion than the low ac4Cscore group (Figures 3F,G). For the high ac4Cscore group, the most significant peak of amplification fell in the cytoband of 8q24.21. Genes located in this region, including *MYC*, *POU5F1B* and *PVT1*, were notorious for tumor progression. Other peaks fell in 1q21.3 and 17q25.3. The deletion peak fell in the cytobands of 17p13.1, 13q14.2 and 8p23.2 (Figure 3F and Supplementary Table S5, 6). For the low ac4Cscore group, the amplification peaks included 6p21.1, 11q13.3 and 17q25.3, involving *VEGFA*, *CD7*, *CSNK1D* and *UTS2R.* The deletion peaks also contained 17p13.1, 1p36.31 and 13q14.2 (Figure 3G and Supplementary Table S7, 8). The above results show a different distribution of somatic mutations between the two groups.

Meanwhile, differentially expressed genes between the two ac4Cscore groups were determined (Supplementary Table S9). GSEA results illustrated that upregulated genes in the high ac4Cscore group were mainly enriched in the cell cycle and DNA replication pathways (Figures 3H,I), suggesting that patients’ poor prognosis might be caused by the rapid proliferation of tumor cells. The activated ribosome pathway in the high ac4Cscore group was consistent with previous findings that ac4C modification of mRNA might promote translation (Figure 3J), again confirmed the efficacy of our model.

### ac4Cscore is Associated With Tumor Stemness

**FIGURE 4:**
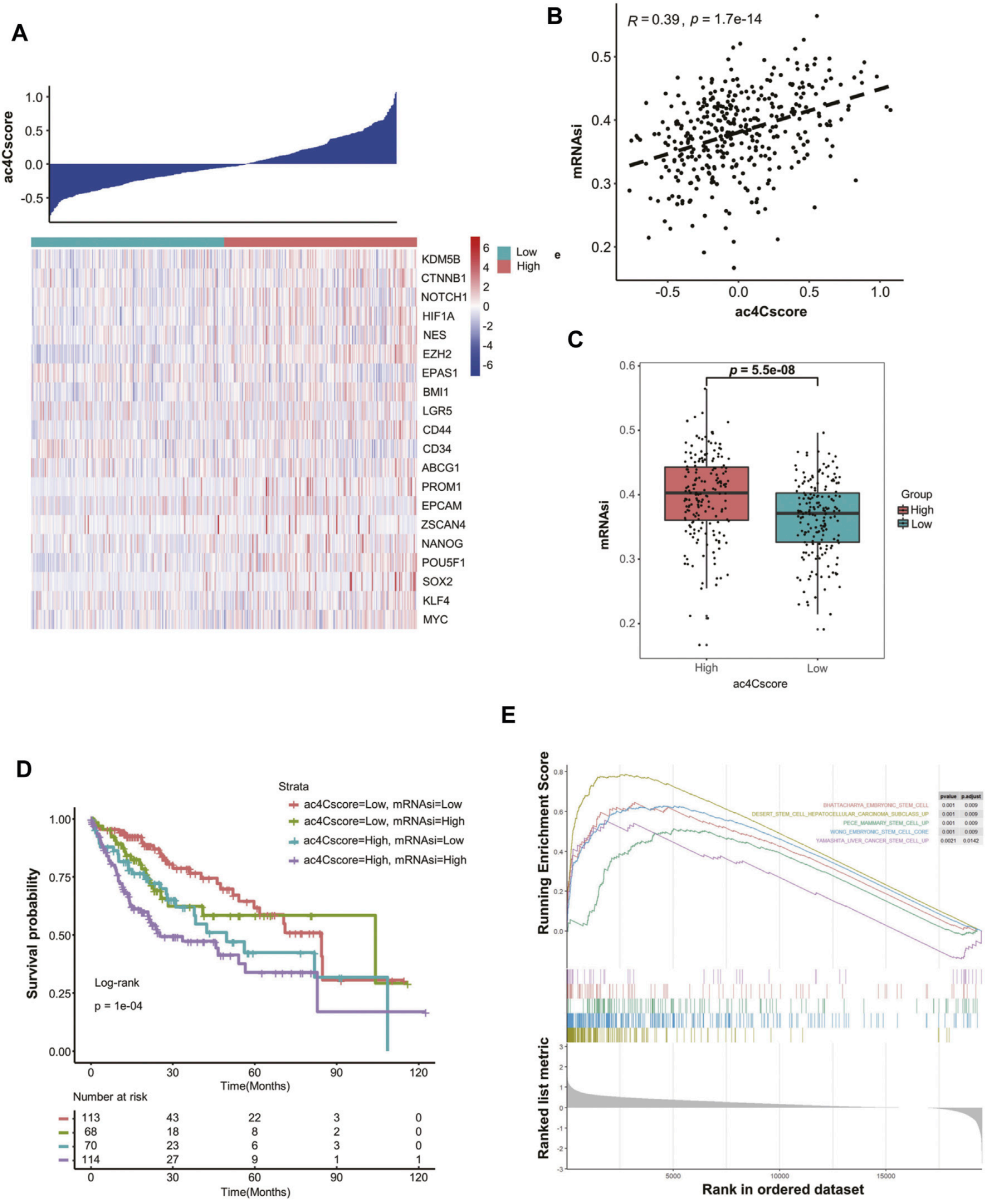
ac4Cscore is associated with tumor stemness. **(A)** Heatmap delineates the relationship between ac4Cscore and stem cell markers. **(B)** Correlation of ac4Cscore and estimated mRNAsi. A Pearson correlation coefficient with *p* value < 0.05 is considered significant. **(C)** The mRNAsi values between the ac4Cscore high and low groups. Each dot represents the value of individual patient. T test with *p* value < 0.05 is considered significant. **(D)** Survival analysis based on ac4Cscore and mRNAsi of patients. Log-rank t test with *p* value < 0.05 is considered significant. **(E)** GSEA plots show the upregulated genes in the ac4Cscore high group enriched in several stemness-related gene sets. Strict criterion with *p* value < 0.01 and adjusted *p* value < 0.05 is considered significant.

To elicit the underlying connection of ac4Cscore with tumor stemness, the expression of several stemness-related genes was extracted. With increased ac4Cscore among patients, the expression of most markers, such as *KDM5B*, *EZH2*, *CD44* and *POU5F1*, tended to be elevated (Figure 4A). A significant correlation between ac4Cscore and these genes was observed (Supplementary Figure S6A–D). However, several markers exhibited no significant ascent or even an opposite trend (Supplementary Figure S6E,F). Considering that single gene expression might be confounded by tumor heterogeneity and tissue sampling bias in bulk analysis, we adopted a machine learning method, termed mRNAsi, to comprehensively analyze the association of ac4Cscore and tumor stemness (Supplementary Table S10). The Pearson correlation coefficient results showed that a significantly positive correlation between ac4Cscore and mRNAsi existed (Figure 4B). In addition, the ac4Cscore-high group had a remarkably higher mRNAsi than the low ac4Cscore group (Figure 4C). We also noticed that the combination of mRNAsi and ac4Cscore could be a prognostic indicator to predict patient outcome. Patients with high ac4Cscore and high mRNAsi had the worst prognosis, while those with low ac4Cscore and low mRNAsi had a better prognosis (Figure 4D). The predictive power was strong within 60 months. Finally, GSEA was performed, and the results demonstrated that various stem cell gene sets were enriched in ac4Cscore-high group (Figure 4E). Moreover, diverse mechanisms, including the base excision repair, mismatch repair and nucleotide excision repair pathways, which could help CSCs survive in extreme environments, were also enriched in ac4Cscore-high group (Supplementary Figure S6G). These findings uncover the association of ac4Cscore and tumor stemness.

### Characteristics of the Tumor Microenvironment in Different ac4Cscore Groups

**FIGURE 5:**
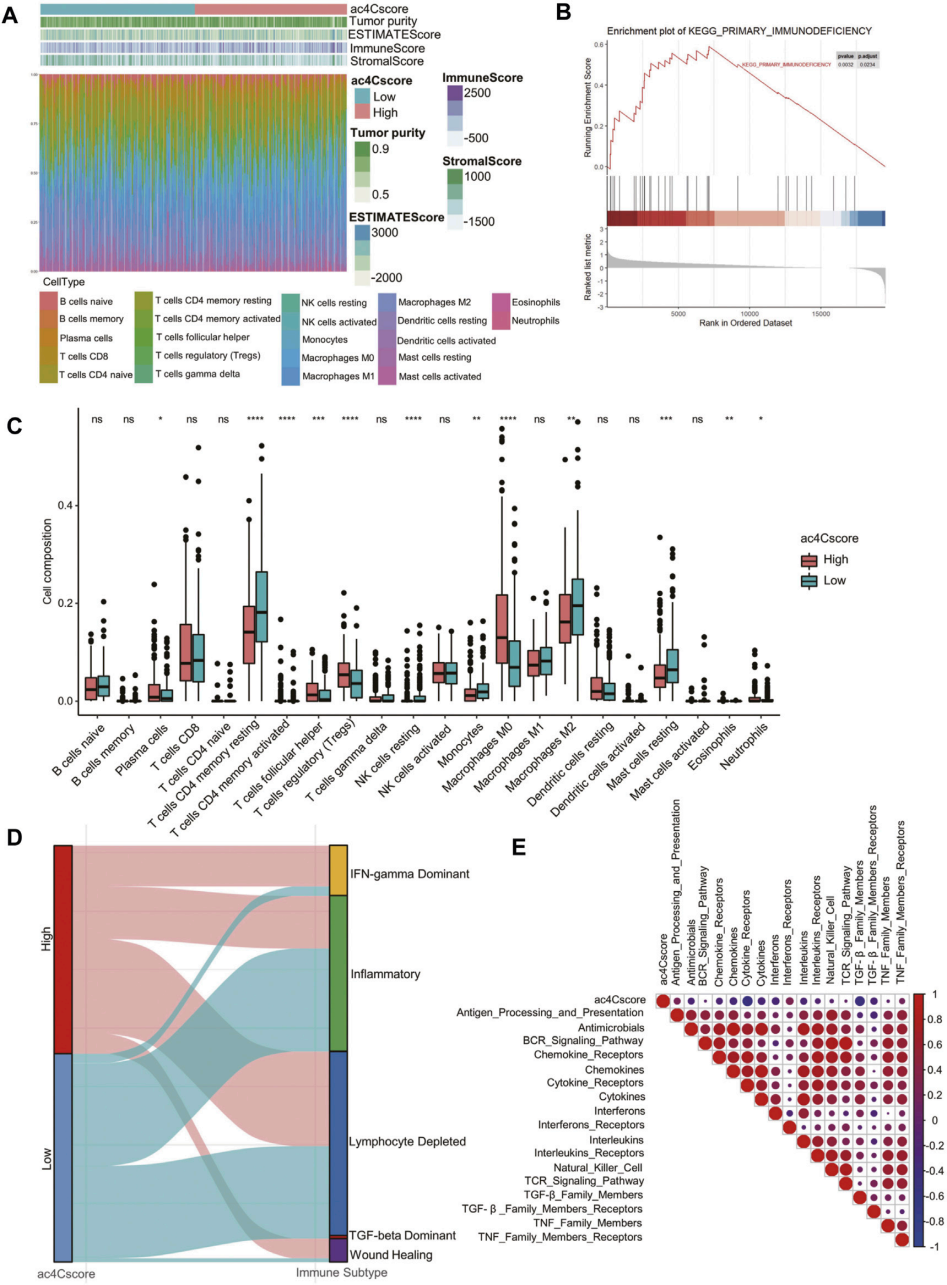
The landscape of the TME and biological characteristics in the ac4Cscore groups. **(A)** The calculated values of the TME score and proportion of immune cell infiltration of each patient. **(B)** GSEA plot shows the upregulated genes in the ac4Cscore high group enriched in the primary immunodeficiency pathway. **(C)** The distribution of 22 immune cell types in different ac4Cscore groups. Wilcoxon test is used for statistical analysis. * *p* < 0.05, ** *p* < 0.01, *** *p* < 0.001, **** *p* < 0.0001 and ns indicates not significant. **(D)** Alluvial diagram depicts the attribution of immune subtypes in the ac4Cscore groups. **(E)** Correlation plot displaying the relationships between ac4Cscore and immune signatures. Pearson correlation coefficient is used for analysis. Size and colors of circle indicates the coefficient.

To quantitatively profile the landscape of the tumor microenvironment in different ac4Cscore groups, both ESTIMATE and CIBERSORT algorithms were performed (Figure 5A and Supplementary Table S11, 12). Upon comparison with the low ac4Cscore group, stromal scores were significantly lower in the ac4Cscore-high group, while immune scores were higher (Supplementary Figure S7A). There were no obvious differences in ESTIMATE scores or tumor purity between the two groups (Supplementary Figure S7B). GSEA results indicated that the primary immunodeficiency pathway was enriched in ac4Cscore-high group, suggesting that the poor prognosis of this group could be caused by immune cell dysregulation (Figure 5B). In this context, we further investigated the differences in immune infiltration of these two groups. The proportions of plasma cells, T cells follicular helper, T cells regulatory, macrophage M0 and neutrophils were more abundant in the high ac4Cscore group, while the proportions of T cells memory resting, NK cells resting, monocytes, macrophage M2 and mast cells resting were more adequate in the low ac4Cscore group (Figure 5C). To better illustrate the role of ac4Cscore in immunity, we extracted available immune subtypes of LIHC. Subtype IFN-gamma Dominant and Wound Healing were mainly consisted of the ac4Cscore-high group, while the low ac4Cscore group was mostly made up of subtypes Inflammatory and TGF-beta Dominant (Figure 5D). Even though subtype Lymphocyte Depleted comprised approximately half of each group, the survival curve showed that the prognosis of patients with higher ac4Cscore was worse than that of the low group (Supplementary Figure S7C). Next, GSVA was conducted to investigate the correlation of ac4Cscore and immune signatures (Supplementary Table S13). The high ac4Cscore group had higher variation of pathway activities in interferon receptors as well as antigen processing and presentation, while higher activity variation of cytokine receptors and TGF-beta family members was observed in the ac4Cscore-low group (Supplementary Figure S7D). Corrplot results showed a positive or negative correlation between ac4Cscore and immune signatures (Figure 5E). The results above suggest that ac4Cscore might have an impact on the tumor microenvironment, especially immune infiltration.

### Validation of ac4Cscore in Other Datasets

**FIGURE 6:**
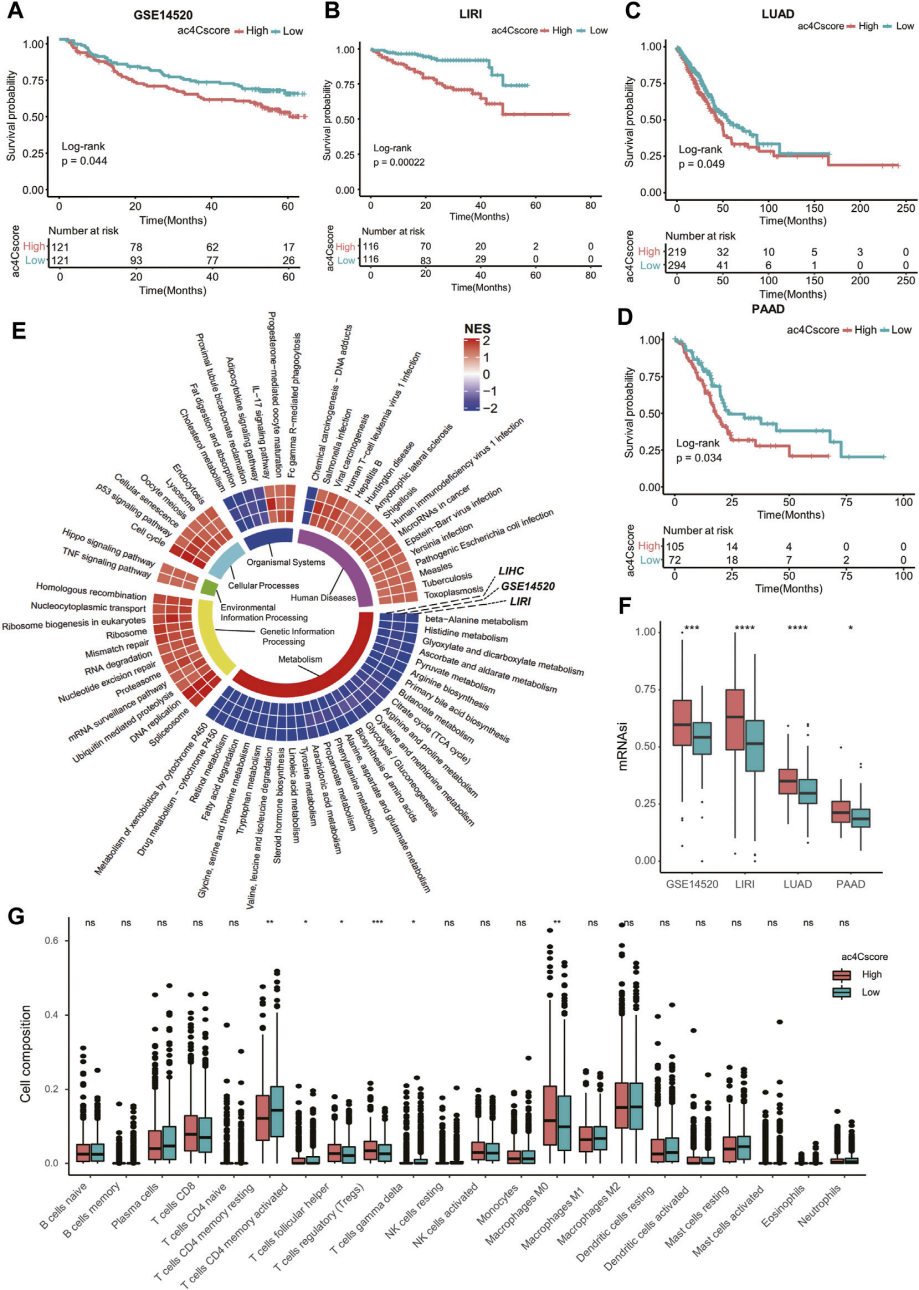
ac4Cscore is a common predictive marker in other datasets. **(A–D)** Survival analysis of the ac4Cscore groups in GSE14520, LIRI, LUAD and PAAD. Kaplan-Meier curves with log-rank *p* values < 0.05 are considered significant. **(E)** Ring heatmap of GSEA-based KEGG pathway analysis between the ac4Cscore-high and ac4Cscore-low groups. The inner most ring annotates pathway categories. Cells in the outer rings are colored by normalized enrichment score (NES) calculated by GSEA. A higher NES means higher pathway activity in the ac4Cscore-high group. Only pathways that showed consistent results in three HCC datasets are extracted and visualized. **(F)** The mRNAsi values for ac4Cscore high and low groups in GSE14520, LIRI, LUAD and PAAD. Dots represent the outliers. **(G)** The distribution of 22 immune cell types for the four ac4Cscore groups. Wilcoxon test is used for statistical analysis. * *p* < 0.05, ** *p* < 0.01, *** *p* < 0.001, **** *p* < 0.0001 and ns means not significant.

To validate the practical value of ac4Cscore, four other datasets were involved in this study. Patient information is listed in Supplementary Table S14. According to the ac4Cscore calculated by a previously described workflow, patients in each dataset were dichotomized into high and low groups. The prognosis of patients from the high ac4Cscore group was significantly worse than that of the low ac4Cscore group (*p* = 0.044 for GSE14520, *p* = 0.00022 for LIRI, *p* = 0.049 for LUAD and *p* = 0.034 for PAAD) (Figures 6A–D). Consistent GSEA results from three HCC cross-datasets for KEGG pathway analysis were extracted. The ac4Cscore-high group showed common downregulation of various metabolic pathways. Of note, cross-dataset results confirmed the upregulation of cell proliferation-related pathways, including DNA replication, cell cycle, Hippo signaling pathway and p53 signaling pathway (Figure 6E). Then, mRNAsi was estimated to represent the tumor stemness of each patient (Supplementary Table S15). The mRNAsi of the high ac4Cscore group was significantly higher than that of the low ac4Cscore group in the four other datasets respectively (Figure 6F). In addition, compared with the low ac4Cscore group, the high ac4Cscore group had a higher mean mRNAsi, and a higher positive correlation with ac4Cscore was also observed (Supplementary Figure S8A). Interestingly, pathways such as nucleotide excision repair, mismatch repair and homologous recombination, which boost CSC survival, were generally upregulated in the ac4Cscore-high group (Figure 6E). Next, the fraction of 22 immune cells of every patient from the four other datasets was predicted (Supplementary Table S16) and merged into high and low ac4Cscore groups. Distinct compositions of T cell types, including CD4 memory resting, CD4 memory activated, follicular helper, regulatory and gamma delta were observed between ac4Cscore high and low groups (Figure 6G). Detailed information about the correlation of the immune cells with ac4Cscore in each group and each dataset is also displayed (Supplementary Figure S8B). In addition, the cross-dataset results also showed consistent upregulation of various immune pathways, such as the IL-17 signaling pathway and Fc gamma R-mediated phagocytosis (Figure 6E). In conclusion, the predictive power of ac4Cscore in patient prognosis, tumor stemness and immune infiltration was confirmed using multiple datasets. The results suggest that ac4Cscore also work in other cancer types, including lung cancer and pancreatic cancer.

### Role of ac4Cscore in Anti-PD1/mTOR Treatment Cohorts

**FIGURE 7:**
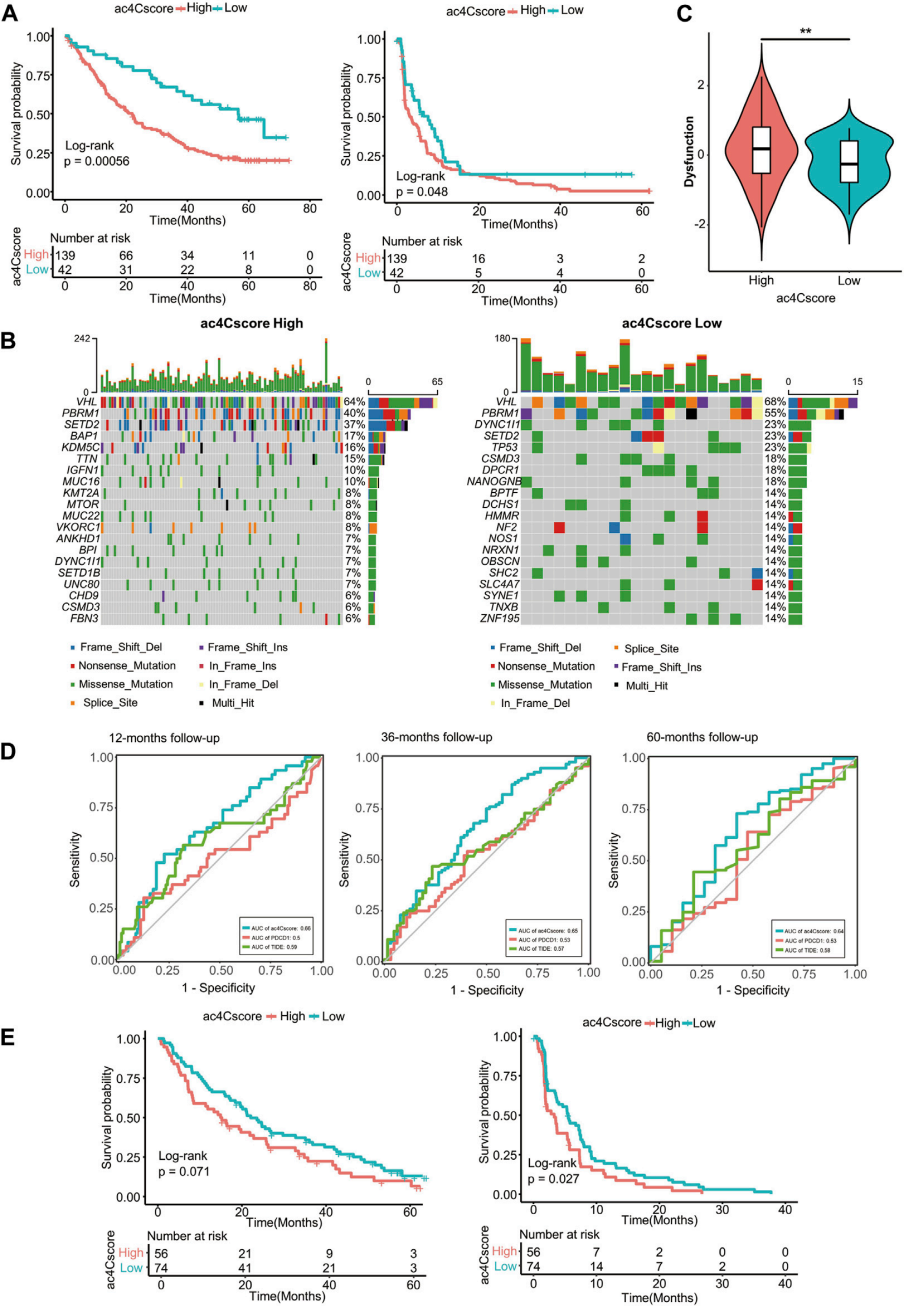
ac4Cscore is a prognostic biomarker in anti-PD1/mTOR treatment cohorts. **(A)** Survival analysis for ac4Cscore groups treated with anti-PD1 inhibitors. Left: overall survival; Right: progression-free survival. **(B)** Waterfall plots depict SNVs and INDELs of patients in the anti-PD1 cohort. Left, ac4Cscore high group; Right, ac4Cscore low group. **(C)** The value of the calculated dysfunction score for the ac4Cscore high and low groups in the anti-PD1 cohort. ** represented *p* < 0.01. **(D)** Roc curves delineating ac4Cscore and the expression of *PDCD1* and TIDE on the overall survival of the anti-PD1 cohort at the 12-, 36- and 60-month follow-ups. **(E)** Survival analysis for ac4Cscore groups treated with anti-mTOR inhibitors. Left: overall survival; Right: progression-free survival.

Existing evidence has shown that the NAT10-mediated ac4C modification pattern is associated with tumor stemness and immune cell infiltration. Therefore, we investigated whether ac4Cscore could indicate patient response to immune checkpoint blockade therapy or targeted treatment with anti-PD1 and anti-mTOR cohorts. Patient information is listed in Supplementary Table S14. In the anti-PD1 cohort, patients with low ac4Cscore presented significant clinical benefits and notably prolonged overall and progression-free survival (Figure 7A). Compared with the low ac4Cscore group, the high ac4Cscore group had elevated mRNAsi (Supplementary Figure S9A and Supplementary Table S15). Different distributions of immune cells could also be observed in these groups (Supplementary Figure S9B and Supplementary Table S16). In addition, the low ac4Cscore group exhibited a more intensified TMB than the high ac4Cscore group (Supplementary Figure S9C), with a mutation rate of 14 versus 6% for the 20th most mutated gene (Figure 7B). Moreover, the TIDE method was adopted to assess tumor immune evasion. Notably, although the two ac4Cscore groups showed distinct outcomes, there were no significant differences in either TIDE score or exclusion score between these groups except dysfunction score (Figure 7C and Supplementary Figure S9D,E), indicating that ac4C mRNA modification should be a novel mechanism involved in T cell dysfunction-related tumor immune evasion and has not been fully considered thus far. The predictive power of ac4Cscore on patient survival was also examined. ROC curves showed that ac4Cscore could be a better indicator than *PDCD1* expression or TIDE score to predict patient outcome at 12, 36 and 60 months (Figure 7D). For those treated with mTOR inhibitors, high ac4Cscore patients suffered from worse overall and progression-free survival than low ac4Cscore patients (Figure 7E). The gene mutation landscape of these two groups was also drawn (Supplementary Figure S9F), illustrating that the high ac4Cscore group had a higher mutation rate of *PBRM1* and *BAP1* than the low ac4Cscore group. The results above suggest that ac4Cscore could be a novel marker to predict patient prognosis with immunotherapy and targeted therapy.

## Discussion

Increasing evidence suggests that posttranscriptional mRNA modification plays an essential role in tumor initiation and progression, raising our attention to the epitranscriptome at an unprecedented level (Bauer et al., 2016; Gu H. et al., 2020; Gu L. et al., 2020; Blanco et al., 2021). As an ancient and highly conserved RNA modification in all domains of life, the process, distribution and function of ac4C in mRNAs has been unraveled in the past few years (Arango et al., 2018; Sas-Chen et al., 2020). However, ac4C remains relatively unexplored, and few studies have focused on how ac4C regulates and influences the intrinsic and extrinsic characteristics of tumors. Here, we reported an ac4Cscore model that can stratify patients into two groups with distinct prognoses caused by different somatic mutation landscapes, stemness features and TME infiltration. Through multiple validation cohorts, we confirmed the conserved efficacy of ac4Cscore in predicting the prognosis of patients (Supplementary Figure S10).

In this study, we obtained ac4C-DEGs comparing HCC with normal liver samples by comprehensive data mining and bioinformatic analysis. In addition, we constructed a predictive model named ac4Cscore by multivariate Cox regression and LASSO algorithms to determine genes with the greatest effect on patient outcome. Multivariate analysis has the advantage of gleaning authentic associations between variables rather than focusing on any specific one. LASSO regression computes the average error and standard deviation over each fold and fits a generalized linear model by maximizing the partial likelihood with an elastic net penalty. These two methods enabled the selection of optimal genes, including a favorable factor, *COL15A1*, and two risk factors, *G6PD* and *TP53I3*. In other words, ac4C-related modification genes are a double-edged sword that inhibit and promote tumors at the same time. The ac4Cscore model managed to dichotomize patients into two groups. Of note, the low ac4Cscore group had a significantly prolonged survival outcome compared with the high ac4Cscore group. We also observed that pathologically, the high ac4Cscore group consisted of patients with advanced tumor grade. In this context, we sought to determine the characteristics of tumors behind ac4C modification patterns. For somatic mutation profiles, the SNVs and INDELs rate of *TP53* ranked the most frequently in the high ac4Cscore group, much higher than that in the low ac4Cscore group. The loss of this suppressor gene activation is strong evidence for the poor prognosis of patients (Staib et al., 2003). Another proof supporting our findings was the amplification regions of the high ac4Cscore group, involving cytoband 8q24.21, where oncogenes *MYC*, *POU5F1B* and lncRNA *PVT1* are located. The promoting roles of these genes in HCC have been widely studied and revealed (Qu et al., 2014; Pan et al., 2018; Jiang et al., 2020). We then performed GSEA to uncover the concordant biological difference between the two groups and observed that the cell cycle and DNA replication were enriched in the high ac4Cscore group, which is responsible for rapid tumor growth. We also found that the ribosome pathway was highly activated in the high ac4Cscore group. This made sense because Arango et al proved that ac4C modification promoted mRNA translation (Arango et al., 2018).

The ac4Cscore model was made up of three genes, *COL15A1*, *G6PD* and *TP53I3*. Collagen type XV alpha one chain (*COL15A1*) is a protein-coding gene mainly localized at basement membrane zones and is essential for maintaining the structure of the extracellular matrix. Emerging evidence has demonstrated that it is secreted from a fibroblast-tumor cell hybrid and acts as a tumor suppressor. Re-expression of *COL15A1* in the HeLa cell line hindered tumor formation in a xenograft model (Mutolo et al., 2012), and the ability of *COL15A1* to stabilize the extracellular matrix also prevented distal tumor metastasis (Clementz and Harris, 2013). Glucose-6-phosphate dehydrogenase (*G6PD*) is a housekeeping gene located on the X chromosome. It encodes a cytosolic enzyme whose main function is to produce nicotinamide adenine dinucleotide phosphate (NADPH) and maintain redox homeostasis. Evidence has shown that *G6PD* is essential for cell growth, survival and embryonic development through redox-sensitive mechanisms and is therefore upregulated in several tumor types (Yang H. C. et al., 2019). In addition, the G6PD-NADPH redox system was shown to be important for stabilizing the metabolism of T cells and regulating antitumor immunity (Gu et al., 2021). It was reported as a prognostic marker to predict the immunotherapy response of Merkel cell carcinoma (Nakamura et al., 2020). For tumor protein p53 inducible protein 3 (*TP53I3*), it functions as reactive oxygen species and involves in DNA damage response pathway (Kotsinas et al., 2012). There were experiments illustrating oncogenic role of *TP53I3* in papillary thyroid cancer through activating the PI3K/AKT/PTEN pathway (Xu et al., 2015) and in non-small cell lung cancer by promoting mitotic progression (Li et al., 2017). In summary, the ac4Cscore model was formed from three key genes that individually had proven physiological functions in tumors, thereby representing ac4C mRNA modification patterns.

Except for cancer cells, the TME contains CSCs, stromal and immune cells, and other molecular components that interact with cancer cells and contribute to the development of tumors. A recent review suggested that CSC-immune cell crosstalk impacted tumor growth and the immune response, revealing their synergistic effect on immune escape, therapeutic resistance and recurrence (Bayik and Lathia, 2021). Despite the scarce evidence showing a direct association of ac4C with the TME, it is reasonable to hypothesize that ac4C regulates tumor stemness and immune infiltration. In this context, we examined ac4Cscore with such tumor characteristics. Cancer stem cells drive tumorigenesis, eventually leading to tumor progression and therapy resistance (Batlle and Clevers, 2017). We found that ac4Cscore was positively correlated with tumor stemness, and some stem cell-related gene sets were enriched in the high ac4Cscore group. The DNA repair system plays a key role in maintaining genome stability. However, they also confer the ability of CSCs to survive in extreme conditions by promoting the DNA damage sensor and repair machinery (Maugeri-Sacca et al., 2012). Additionally, we quantitatively calculated the infiltration of noncancerous cells. Since being proposed, the ESTIMATE and CIBERSORT algorithms have been proven effective in assessing the presence of stromal and immune cells. Evidence has shown the antitumor immunity of regulatory T helper cells, and diverse macrophages boost tumor progression and metastasis (Qian and Pollard, 2010; Togashi et al., 2019). We observed different stromal scores and immune scores along with the distribution of T cells and macrophages, which together assisted tumor evasion of immune destruction, accounting for the distinct prognosis of patients in the two ac4Cscore groups. Notably, the ac4Cscore high group predominated in the immune subtypes IFN-gamma Dominant and Wound Healing, which were classified as having increased expression of angiogenic genes and a higher proliferation rate, while the ac4Cscore low group was mainly attributed to subtype Inflammatory, defined as low to moderate tumor cell proliferation (Thorsson et al., 2018). These intrinsic and extrinsic characteristics of tumors explained the different outcomes of patients in the two ac4Cscore groups. Finally, we validated the results in different primary tumor cohorts, suggesting the predictive power of the ac4Cscore model. Considering this, we next analyzed clinical cohorts treated with anti-PD1 and anti-mTOR inhibitors respectively. We also noted worse overall survival and progression-free survival in the high ac4Cscore group in both cohorts. Specifically, in the anti-PD1 cohort, we observed a more extensive TMB status in the low ac4Cscore group. Accumulating evidence has demonstrated that patients with high TMB correlate with an enhanced response, benefit from immunotherapy and therefore have prolonged survival (Goodman et al., 2017). Conventionally, the expression level of *PDCD1* predicts the outcome of anti-PD1 treatment. Recently, TIDE has emerged as a novel biomarker for predicting the outcome of melanoma and non-small lung cancer patients. The dysfunction score, instead of the TIDE score or exclusion score, which was calculated by TIDE, was significantly different between the ac4Cscore groups. This result suggested that ac4C mRNA modification related genes might impact T cell dysfunction with higher infiltration of cytotoxic T cells, such as T regulatory cells, but was not associated with the immune exclusion microenvironment. In comparison, time-dependent ROC curves showed that ac4Cscore was better than the expression of PDCD1 or TIDE in predicting the OS of patients. These results indirectly supported the crucial role of the ac4Cscore model.

In conclusion, we developed an ac4Cscore model and proved that it was a promising biomarker for the prediction of patient outcome. The easy-to-use ability of ac4Cscore because it consists of only three genes broadens its clinical application. Our study showed for the first time that ac4C-related mRNA modification patterns might play an important role in regulating the intrinsic and extrinsic characteristics of tumors via a plethora of pathways, thus influencing the prognosis of patients. We complemented the role of transcriptional mRNA modification in tumor initiation and progression. However, limitations still remain. Our findings were based on bulk RNA sequencing data, further analysis at single cell resolution could provide more rigorous information. Considering that tumors are a complicated system, the accuracy of this model is not outstanding, and how ac4C modification patterns directly impact the TME needs further experimental confirmation.

## Data Availability

The original contributions presented in the study are included in the article/Supplementary Material, further inquiries can be directed to the corresponding author.

## Abbreviations

ac4C, N4-acetylcytidine; CNV, copy number variants; CSCs, cancer stem cells; DEGs, differentially expressed genes; GEO, gene expression omnibus; GISTIC, genomic identification of significant targets in cancer; GO, gene ontology; GSEA, gene set enrichment analysis; GSVA, gene set variant analysis; HCC, hepatocellular carcinoma; ICGC, international cancer genome consortium; INDEL, insertion-deletion; KEGG, kyoto encyclopedia of genes and genomes; LASSO, least absolute shrinkage and selection operator; LUAD, lung adenocarcinoma; MATH, mutant-allele tumor heterogeneity; mRNAsi, mRNA expression-based stemness index; MSigDB, molecular signatures database; OCLR, one-class logistic regression; PAAD, pancreatic adenocarcinoma; ROC, receiver operating characteristic; SNV, single nucleotide variants; TCGA, the cancer genome atlas; TIDE, tumor immune dysfunction and exclusion; TMB, tumor mutation burden; TME, tumor microenvironment; TPM, transcripts per kilobase million.
