# Supplementary material for: Uncovering N4-Acetylcytidine-Related mRNA Modification Pattern and Landscape of Stemness and Immunity in Hepatocellular Carcinoma
Source: Front Cell Dev Biol. 2022 Apr 14;10:861000. doi: 10.3389/fcell.2022.861000 (PMC9046676; doi:10.3389/fcell.2022.861000)
Supplement: Supplementary file 1 [file DataSheet1.PDF]

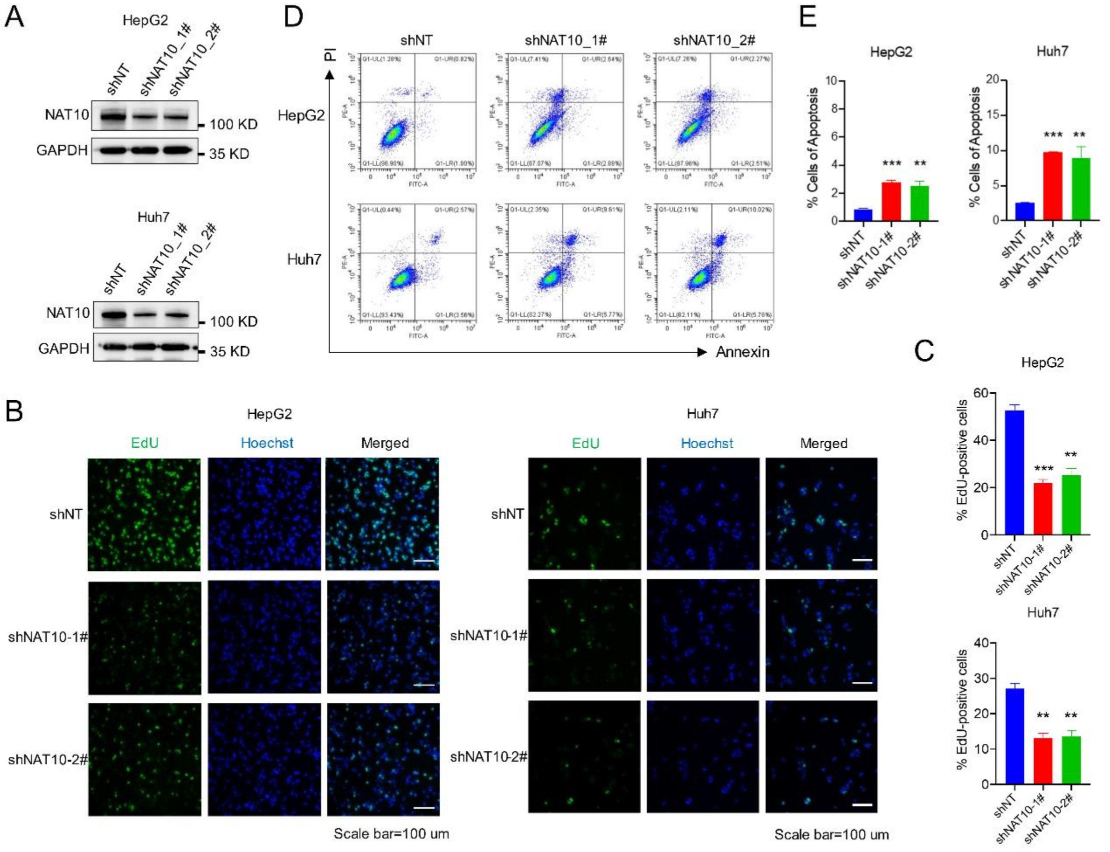

Figure S1. Knocking down NAT10 inhibit HCC cells proliferation and promote apoptosis. (A) Western blot analysis of knockdown NAT10 expression in HepG2 and Huh7 cells. (B-C) Cell proliferation after NAT10 knock down in HepG2 and Huh7 cells was detected by EdU staining (B) and quantitatively analyzed (C) Scale bar, 100 μm. (D-E) Flow cytometer analysis detected apoptosis after NAT10 knockdown in HepG2 and Huh7 cells (D) and quantitatively analyzed (E). **, p<0.01; ***, p<0.001.

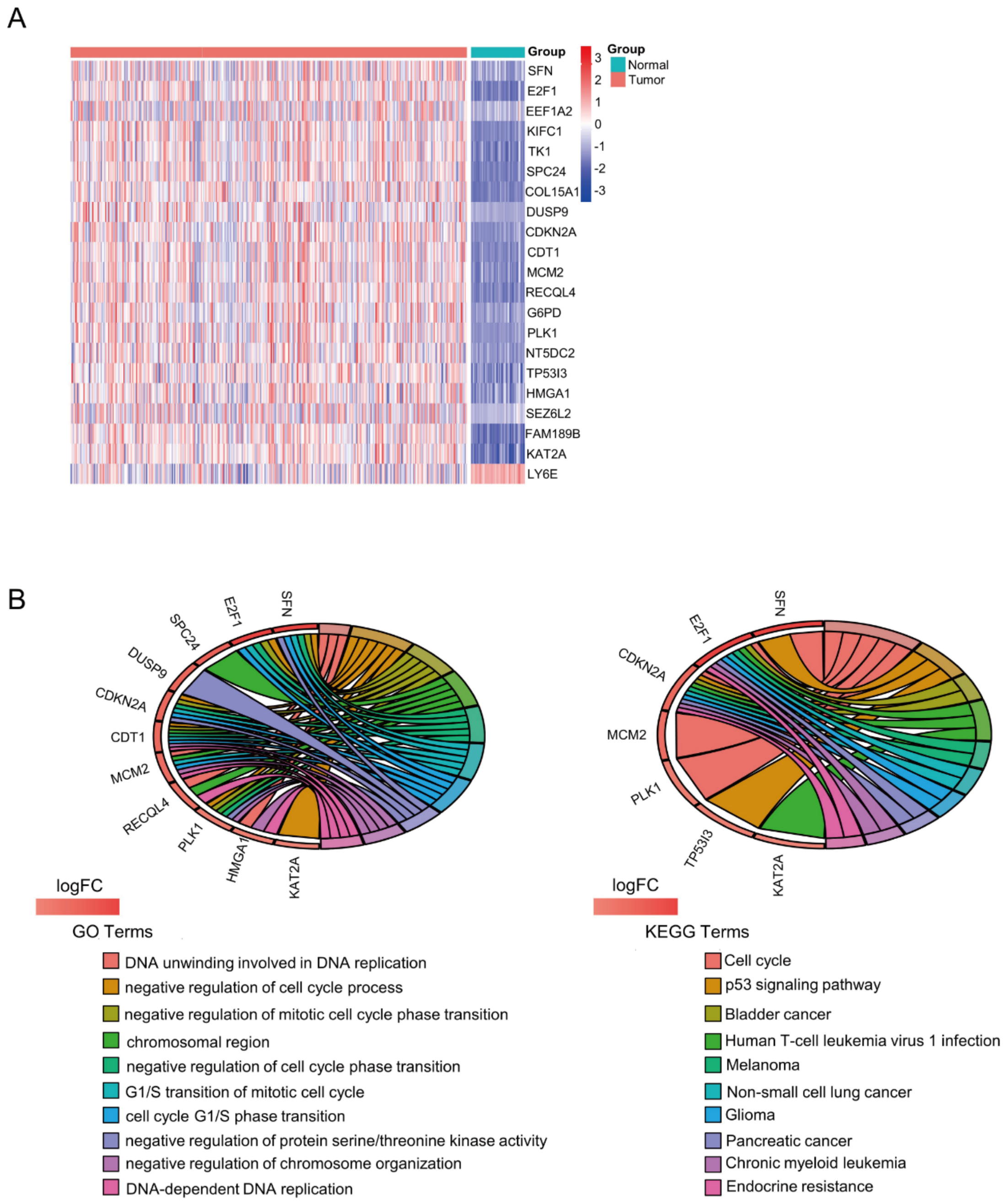

Figure S2. Expression and function of ac4C-DEGs. (A) Heatmap displays the expression of ac4C-DEGs between 368 tumor and 50 normal samples in LIHC. (B) Chord plots show the top ten enriched GO and KEGG terms of ac4C-DEGs.

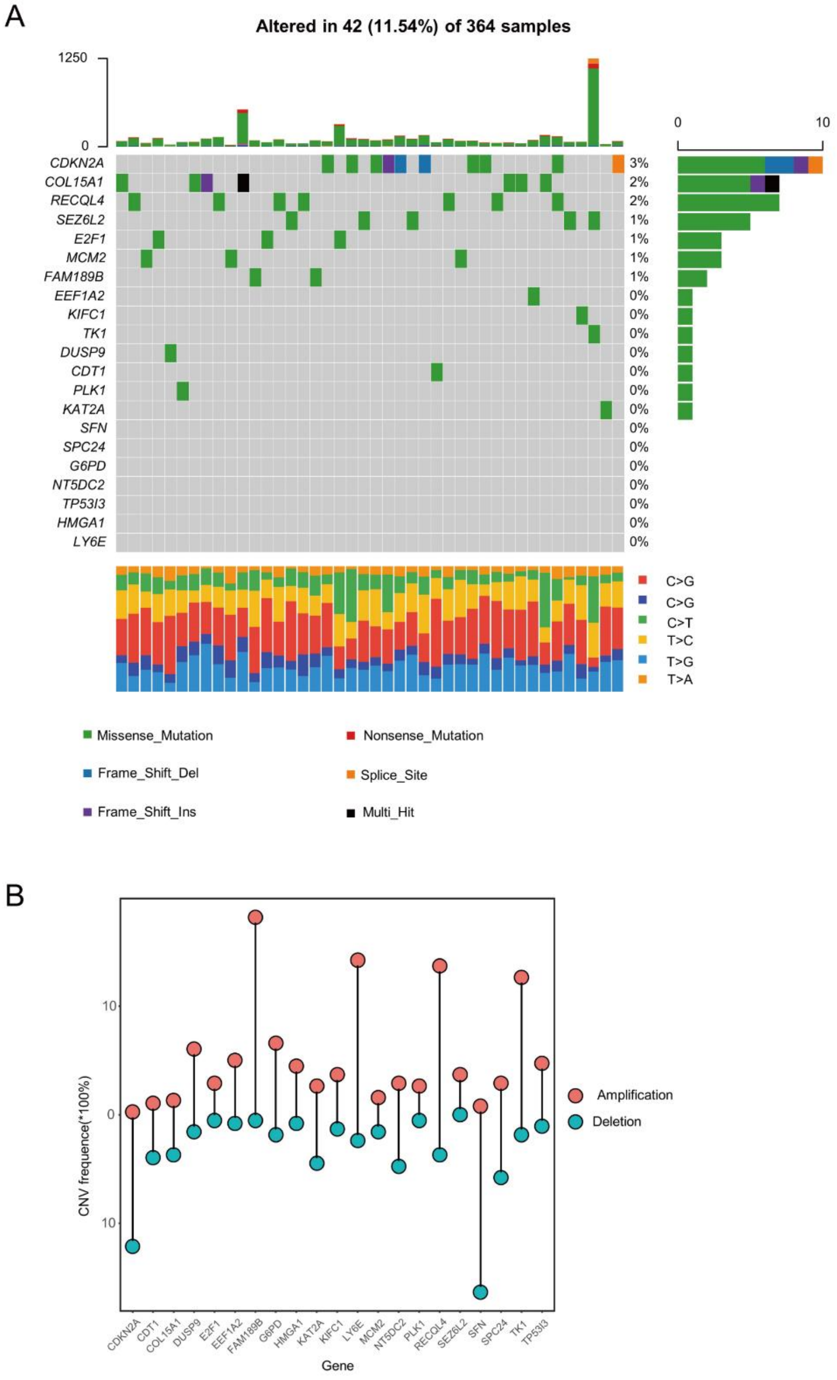

Figure S3. Somatic mutation of ac4C-DEGs in LIHC. (A) The SNV and INDELs of 21 ac4C-DEGs. Each column represents a sample and each row represents a gene. The top panel shows the total mutations in each sample. The bottom panel displays fraction of conversions in each sample. The right number indicates the variation frequency of each gene. The right bar shows the sum of each variant type. (B) The CNV alteration frequency of ac4C-DEGs.

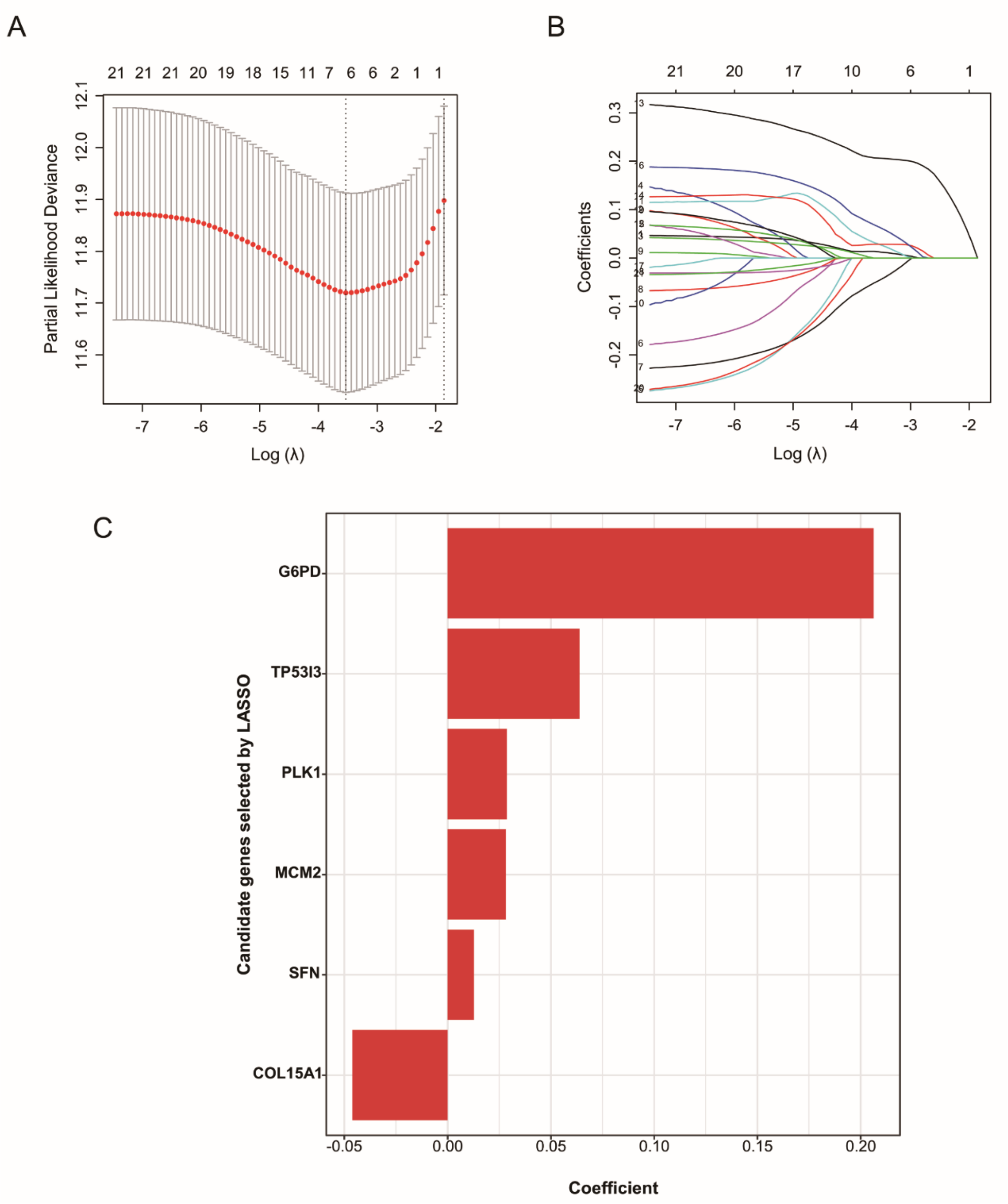

Figure S4. LASSO algorithm for determining the key genes. (A) Plot of the cross-validated error rates. Red dot represents partial likelihood deviance, with error bars showing the standard error. (B) Plot shows the coefficients of 21 ac4C-DEGs regularized with log lambda. (C) The multivariate cox regression model of 6 candidate genes selected by LASSO.

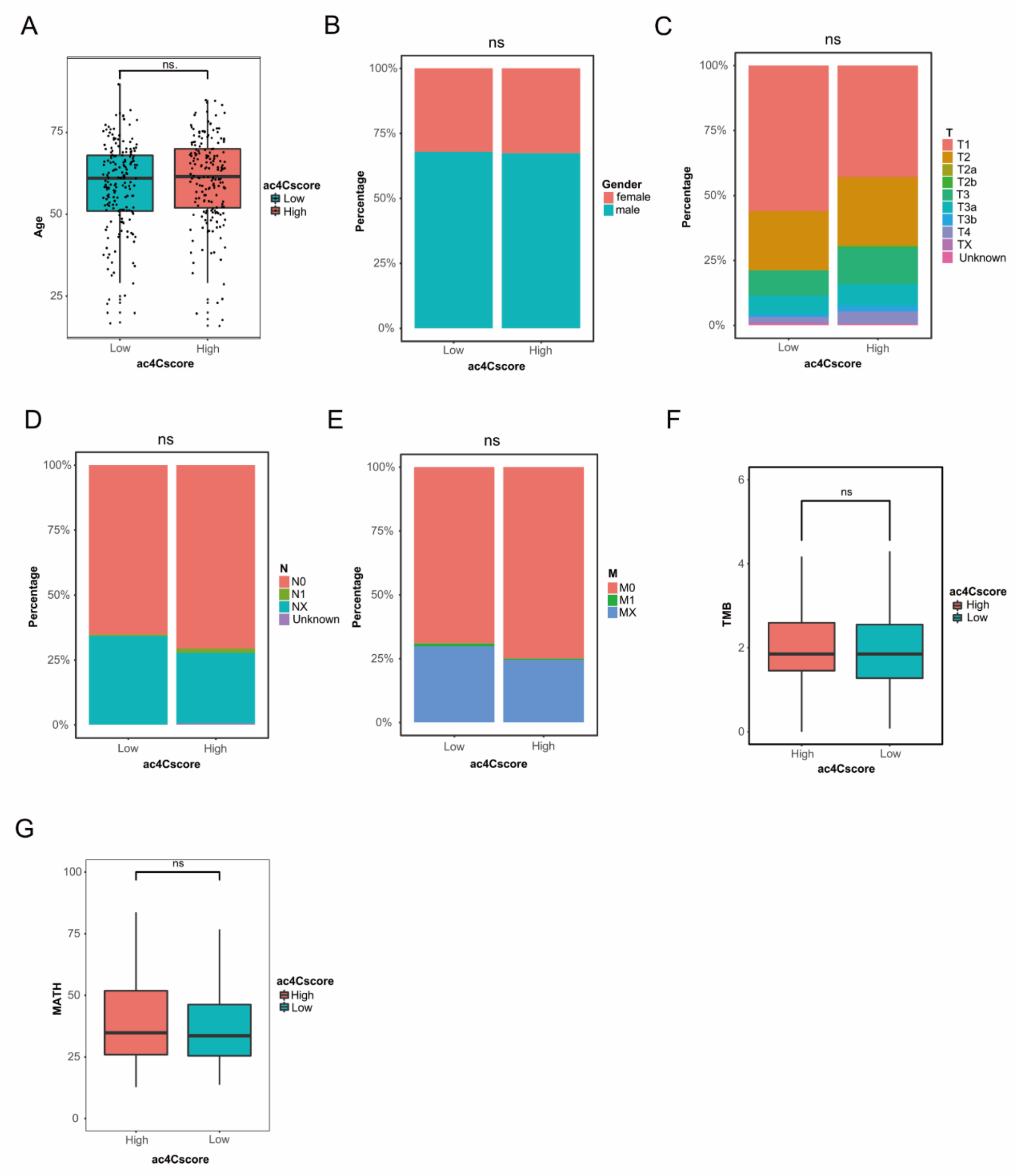

Figure S5. Clinical characteristics of ac4Cscore groups. Plots show the distribution of (A) age, (B) gender, (C) T stage, (D) N stage, (E) M stage, (F) TMB values and (G) MATH values in ac4Cscore high and low groups. T test and fisher's exact test with p value < 0.05 are considered as significant. ns, not significant.

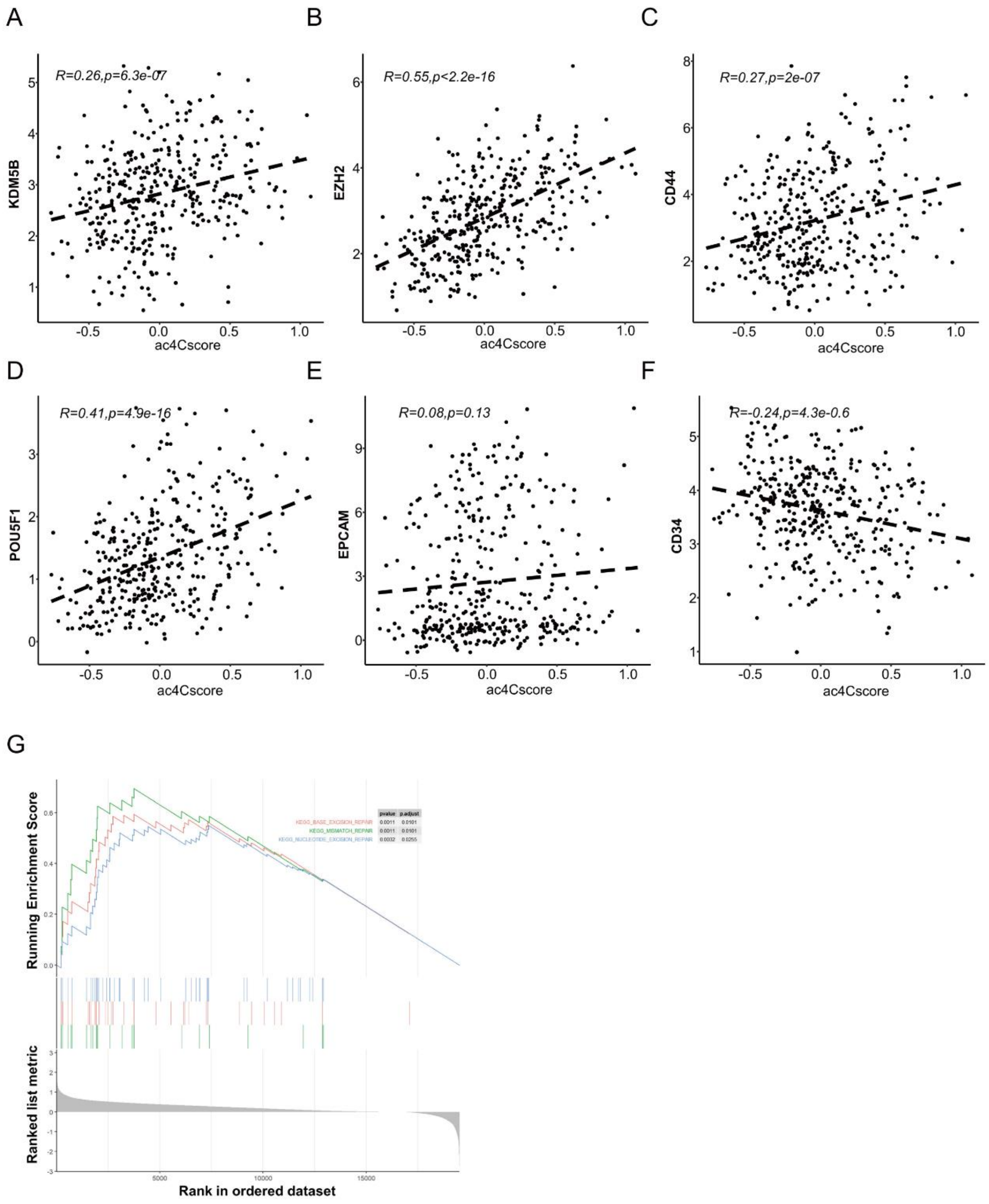

Figure S6. Association of ac4Cscore and tumor stemness. (A-F) Correlation of ac4Cscore and expression of stem cell markers. (G) GSEA plot shows the up-regulated genes in ac4Cscore high group enriched in several DNA repair pathways.

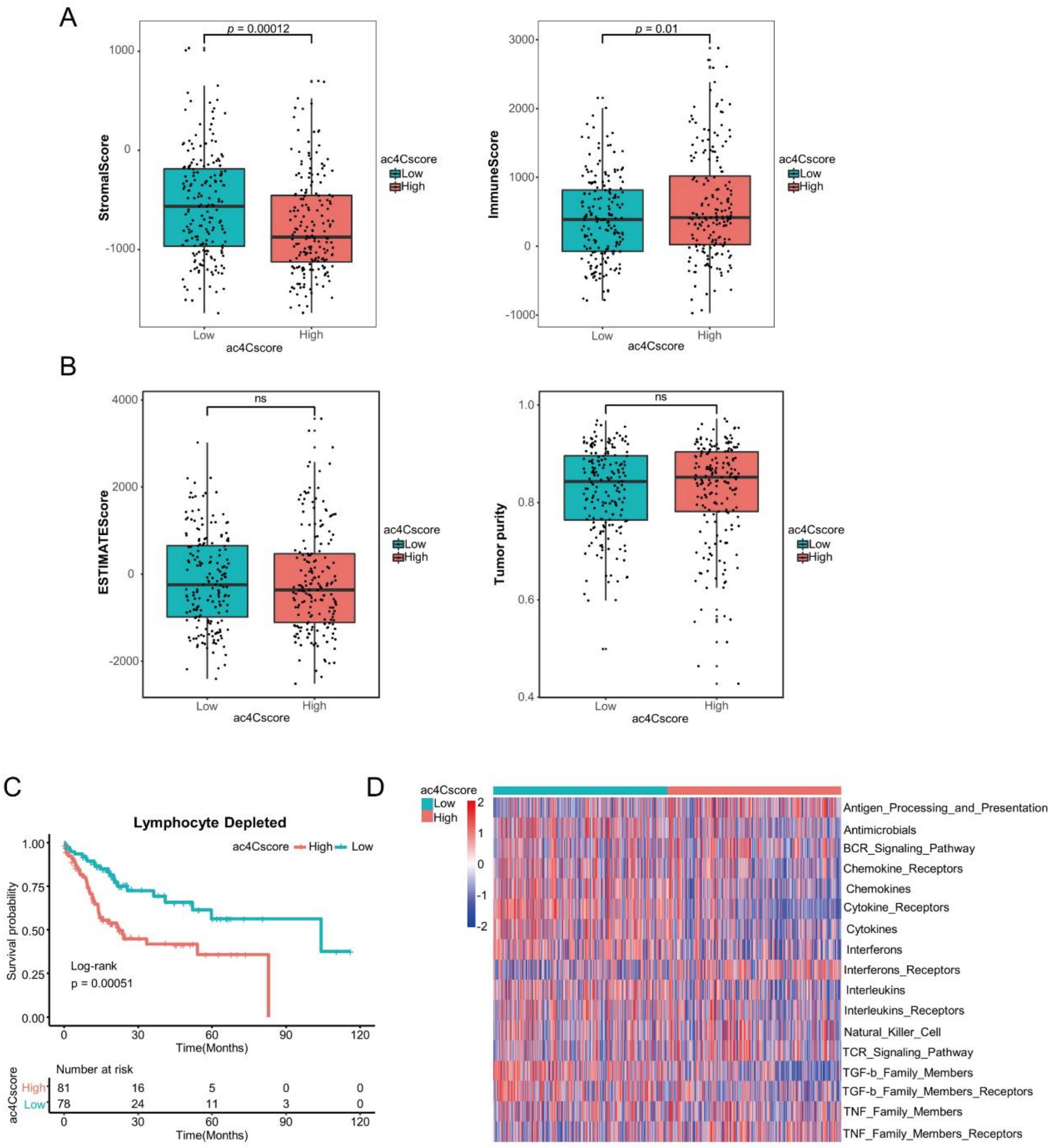

Figure S7. Different characteristics of TME in ac4Cscore groups. (A-B) The calculated values of TME score between ac4Cscore high and low groups. Each dot represents the value of individual patient. T test with p value < 0.05 is considered as significant. NS. Not significant. (C) Survival analysis for immune subtype Lymphocyte Depleted in ac4Cscore groups. Kaplan-Meier curve with log-rank p value < 0.05 is considered as significant. (D) Heatmap shows the relationship between ac4Cscore and immune signatures.

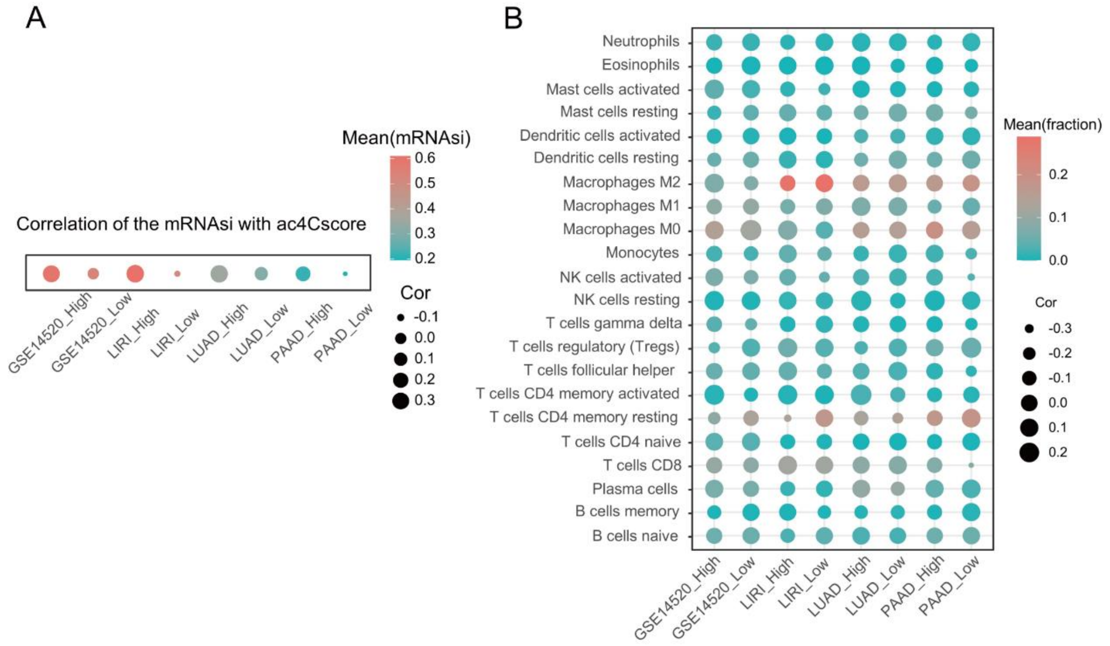

Figure S8. Association of ac4Cscore with mRNAsi and fractions of immune cells in other datasets. (A) Plot shows the correlations between ac4Cscore and mRNAsi, and the estimated mean value for ac4Cscore groups. (B) Plot shows the correlations between ac4Cscore and 22 immune cell types, and the estimated mean fraction of cells for ac4Cscore groups.

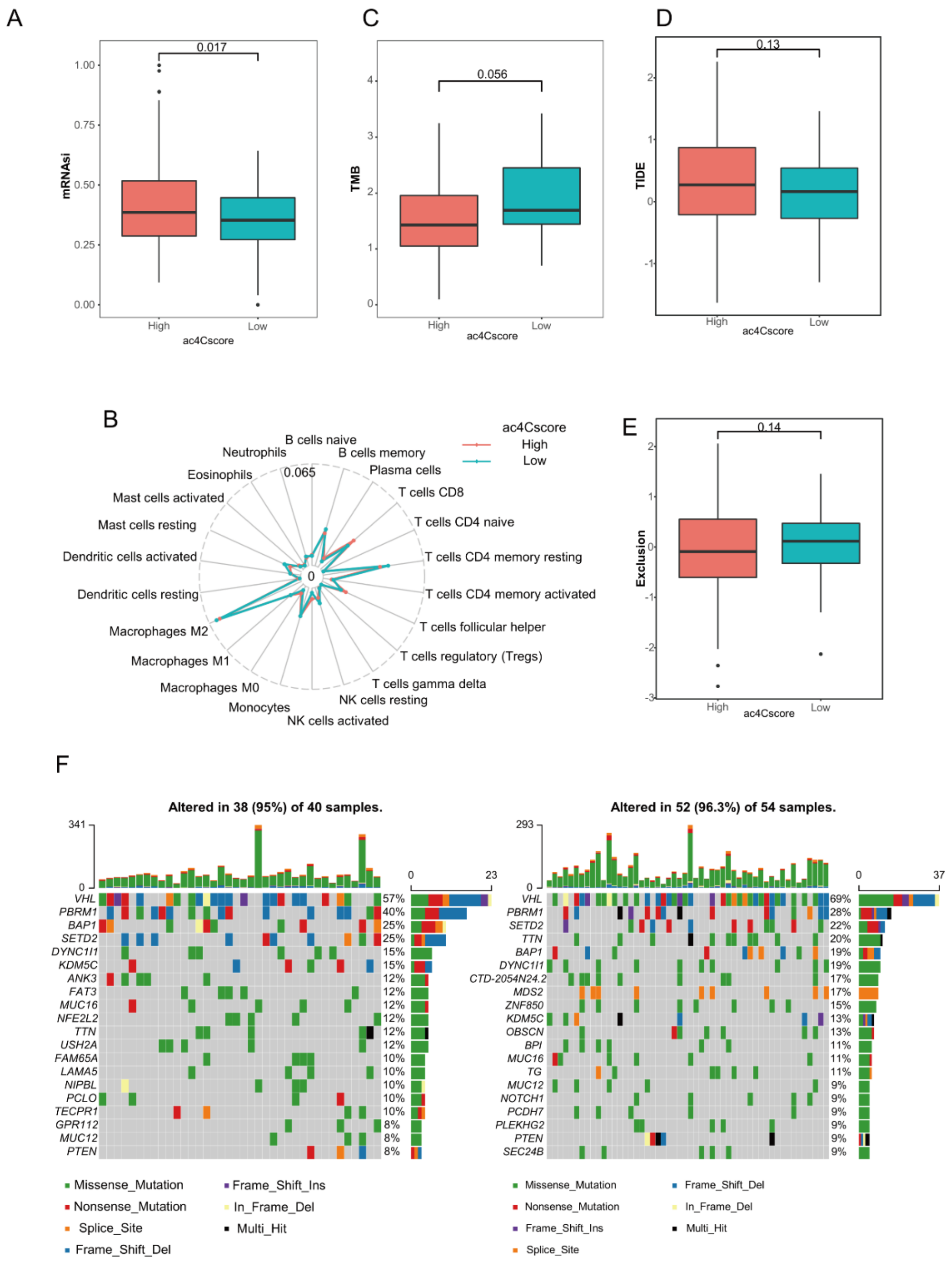

Figure S9. Biological characteristics of ac4Cscore groups in anti-PD1/mTOR treatment cohorts. (A) The values of mRNAsi for ac4Cscore high and low groups in anti-PD1 cohort. Dots represent the outliers. (B) The distribution of 22 immune cell types for ac4Cscore group in anti-PD1 cohort. (C) The value of calculated TMB for ac4Cscore high and low groups in anti-PD1 cohort. (D) The value of calculated TIDE for ac4Cscore high and low groups in anti-PD1 cohort. (E) The value of calculated exclusion score for ac4Cscore high and low groups in anti-PD1 cohort. T test is used for statistical analysis. (F) Waterfall plots depict SNVs and INDELs of patients in the anti-mTOR cohort. Left, ac4Cscore high group; Right, ac4Cscore low group.

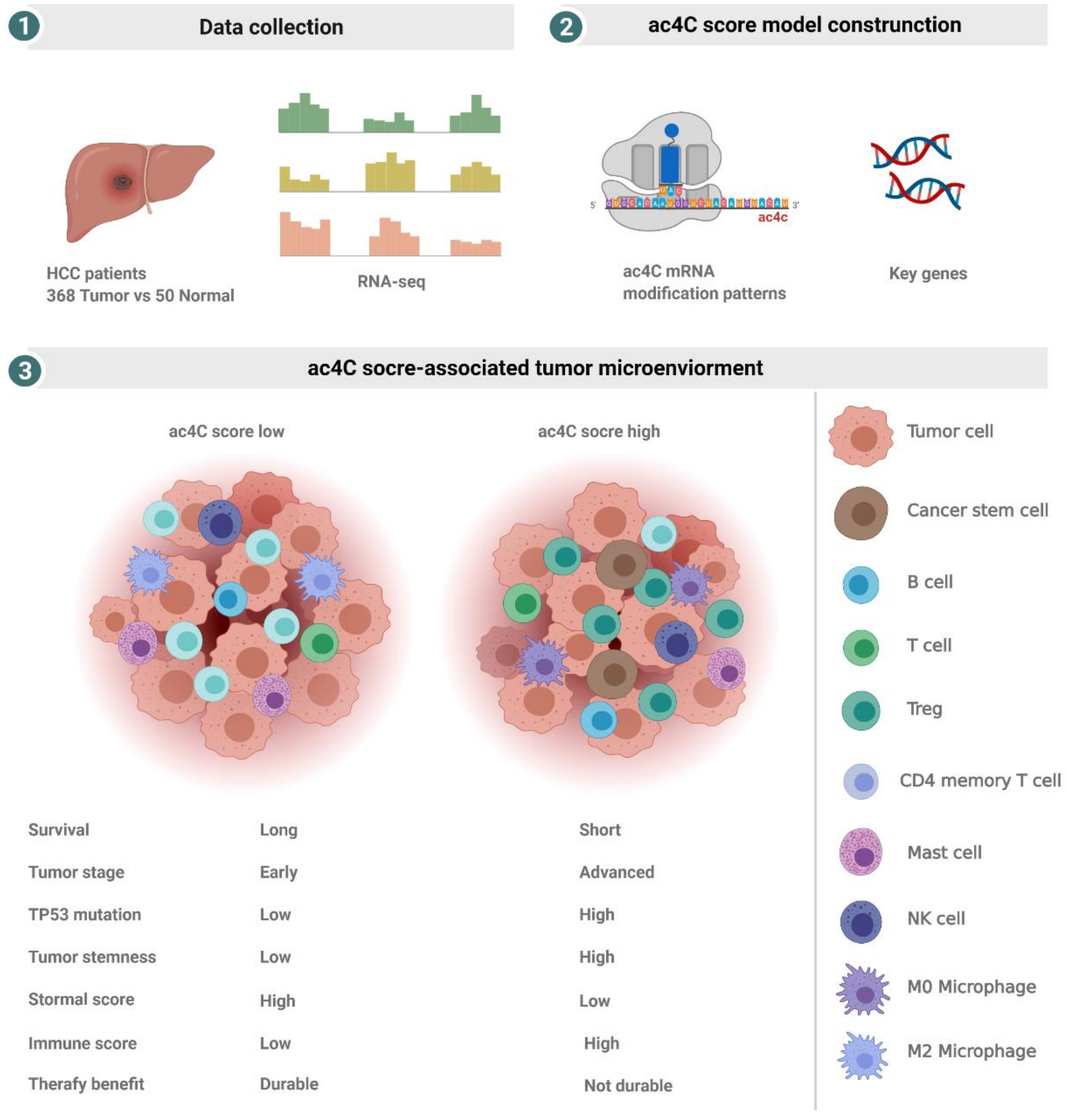

Figure S10. Graphical abstract for construction, prediction and comprehensive characterization of the ac4Cscore groups.
